# Attenuated Salmonella potentiate PD-L1 blockade immunotherapy in a preclinical model of colorectal cancer

**DOI:** 10.3389/fimmu.2022.1017780

**Published:** 2022-12-20

**Authors:** Besan H. Al-Saafeen, Ashraf Al-Sbiei, Ghada Bashir, Yassir A. Mohamed, Razan J. Masad, Maria J. Fernandez-Cabezudo, Basel K. al-Ramadi

**Affiliations:** ^1^ Department of Medical Microbiology and Immunology, College of Medicine and Health Sciences, United Arab Emirates University, Al Ain, United Arab Emirates; ^2^ Department of Biochemistry and Molecular Biology, College of Medicine and Health Sciences, United Arab Emirates University, Al Ain, United Arab Emirates; ^3^ Zayed Center for Health Sciences, United Arab Emirates University, Al Ain, United Arab Emirates

**Keywords:** immune checkpoints inhibitors, PD-L1 blockade, Salmonella typhimurium, immunotherapy, colorectal cancer

## Abstract

The use of immune checkpoint inhibitors to treat cancer resulted in unprecedented and durable clinical benefits. However, the response rate among patients remains rather modest. Previous work from our laboratory demonstrated the efficacy of using attenuated bacteria as immunomodulatory anti-cancer agents. The current study investigated the potential of utilizing a low dose of attenuated *Salmonella typhimurium* to enhance the efficacy of PD-L1 blockade in a relatively immunogenic model of colon cancer. The response of MC38 tumors to treatment with αPD-L1 monoclonal antibody (mAb) was variable, with only 30% of the mice being responsive. Combined treatment with αPD-L1 mAb and *Salmonella* resulted in 75% inhibition of tumor growth in 100% of animals. Mechanistically, the enhanced response correlated with a decrease in the percentage of tumor-associated granulocytic cells, upregulation in MHC class II expression by intratumoral monocytes and an increase in tumor infiltration by effector T cells. Collectively, these alterations resulted in improved anti-tumor effector responses and increased apoptosis within the tumor. Thus, our study demonstrates that a novel combination treatment utilizing attenuated *Salmonella* and αPD-L1 mAb could improve the outcome of immunotherapy in colorectal cancer.

## 1 Introduction

Cancer continues to be a serious public health concern worldwide with nearly 10 million deaths attributed to this chronic disease each year (1). Conventional cancer treatments including chemotherapy and radiotherapy have major limitations due to the lack of tumor specificity, inadequate tissue penetration, significant toxicity and rising resistance. The realization that the major driving force underlying cancer development and progression is linked to a compromised immune system has led to the birth of a new modality to treat cancer using immunotherapeutic approaches, such as antibody-mediated immune checkpoint inhibitors (ICIs), cancer vaccines and adoptive T-cell transfer (2). Despite the promising recent breakthroughs and clinical benefits achieved with immunotherapy, not all patients respond similarly to treatment. Therefore, there is an increasing demand to develop novel strategies to improve the efficacy of immunotherapy.

The identification of several so-called immune checkpoint molecules has revolutionized our understanding of anti-tumor immunity (3). Immune checkpoint molecules induce a series of costimulatory and inhibitory signals that regulate antigen recognition and determine the magnitude of T cell responses. Within the tumor microenvironment, inhibitory ligands and their receptors are predominantly overexpressed on both tumor cells and tumor-infiltrating immune cells (4). The best characterized negative immune regulators include cytotoxic T lymphocyte-associated antigen 4 (CTLA-4) which binds to the costimulatory molecules CD80 (B7-1) and CD86 (B7-2), programmed cell death-1 (PD-1) and its ligand (PD-L1), lymphocyte activation gene-3 (LAG-3) and T cell immunoglobulin and mucin-domain containing-3 (TIM-3) proteins (5–7). The past decade has witnessed unprecedented and durable success with the use of ICIs in treating different types of cancer, including melanoma, non-small cell lung carcinoma (NSCLC), renal cell carcinoma (RCC), lymphoma and mismatch-repair deficient (dMMR) tumors (8, 9). However, only a fraction of cancer patients responds to the treatment in which the overall response rate can be as low as ~20-40% (10, 11). This highlights the need to identify potential means by which the resistance to ICIs can be alleviated in order to improve the clinical outcome.

Different innate and acquired resistance mechanisms have been associated with the reduced anti-tumor efficacy of PD-1/PD-L1 inhibitors and these include: (a) Lack of tumor immunogenicity leading to impaired antigen presentation and T-cell recognition (12–15), (b) poor intratumoral T cell infiltration, activation and differentiation (16, 17), (c) immunosuppressive cellular and soluble components of the tumor microenvironment including MDSCs, Tregs, pro-tumor M2 macrophages and their associated cytokines and chemokines (18–21), in addition to (d) T-cell dysfunction that results from the compensatory upregulation of alternate inhibitory immune checkpoint ligands (22). In order to improve the anti-tumor efficacy of PD-1/PD-L1 inhibitors, combination therapies with other immune checkpoint targeting antibodies, chemotherapy, radiotherapy, and tumor neoantigen vaccine that target resistance mechanisms and enhance the different components of the immune system were utilized and succeeded in treating advanced and poorly immunogenic tumors in pre-clinical and clinical models (23, 24).

Several lines of evidence documented the anti-tumor efficacy of the facultative anaerobic bacteria *Salmonella enterica* serovar Typhimurium (hereafter *S. typhimurium*) against a broad-spectrum of murine tumor models (25–28). *Salmonella-*mediated cancer therapy has unique aspects over conventional treatment modalities in the preferential colonization and proliferation in tumor tissues, immunomodulatory effects and engineering plasticity (29–31). In previous studies from our lab and others, the anti-tumor potential of attenuated *S. typhimurium* was associated with its ability to modulate different immune system components and transform the tumor microenvironment form being immunosuppressive into becoming more immunogenic (32–37). It is well established that *Salmonella* bacteria utilize macrophages as their primary niche in the host (38, 39) and this associated with the activation of several immunomodulatory functions in these cells (40). Our lab demonstrated that treating tumor-bearing mice with attenuated *S. typhimurium* induced phenotypic and functional maturation of tumor-infiltrating myeloid cells and, therefore, inhibited their suppressive capacity (41). These changes were accompanied with the increased infiltration of intratumoral T cells and upregulated expression of MHC class II and Sca-1 on myeloid cells. In addition, other studies documented the role of *Salmonella* in manipulating the intratumoral immune components in favor of tumor inhibition through increasing the infiltration of anti-tumor immune cells, upregulating the expression of pro-inflammatory cytokines and chemokines in addition to its role in transforming the function of certain immune cells (29).

Given that the immunosuppressive nature of the tumor microenvironment is a major obstacle to achieving favorable outcome in treating cancer using PD-1/PD-L1 blockade, we hypothesized that *Salmonella*-induced changes in the tumor tissue could ameliorate the immunosuppressive microenvironment and, therefore, enhance the response rate and therapeutic efficacy of PD-1/PD-L1 blockade. In the current study, our group investigated the anti-tumor potential and immunomodulatory activity of a low dose of attenuated *S. typhimurium* and its capability to improve the therapeutic outcome of antibody-based PD-L1 inhibitors in the MC38 murine colon adenocarcinoma model. Our results demonstrated that attenuated *Salmonella* altered the tumor microenvironment, effectively increasing the immunogenicity of this otherwise relatively immunogenic tumor. Mechanistically, this was achieved through an enhancement in the expression of MHC class II proteins on intratumoral myeloid cells, increasing access by CD4^+^ tumor-infiltrating T cells, and decreasing the percentage of intratumoral T cells that express inhibitory immune checkpoint molecules. Combining attenuated *Salmonella* with PD-L1 blockade improved the overall response rate and inhibited MC38 tumor growth more efficiently compared to monotherapy by further enhancing the infiltration and function of T cells, altering the composition of intratumoral myeloid cells and increasing the number of apoptotic cells. Overall, the findings of this investigation provide insights into utilizing attenuated bacteria to enhance the therapeutic efficacy of cancer immunotherapy.

## 2 Materials and methods

### 2.1 Cell line, bacterial strain and mice

The murine colon adenocarcinoma MC38 cell line was kindly provided by Prof. Jo Van Ginderachter (Vrije University Brussel, Belgium). Cells were maintained in Dulbecco’s Modified Eagle Medium (DMEM) media supplemented with 10% FBS, 100 IU/ml penicillin, 100 IU/ml streptomycin and 50 μg/ml gentamicin (all reagents from Gibco-ThermoFisher Scientific). BRD509E is an *aroA/aroD* auxotrophic mutant strain of *Salmonella enterica* serovar Typhimurium and has been previously described (42, 43). The 50% lethal dose (LD_50_) in normal mice of BRD509E given intraperitoneally is >2x10^6^ CFUs. C57BL/6 mice were obtained from the Jackson Laboratory (Bar Harbor, Maine, USA) and bred in the animal facility of the College of Medicine and Health Sciences, UAE University. For the present studies, male mice were used at 8–12 weeks of age.

### 2.2 Tumor implantation and *in vivo* treatment

The procedure for implanting tumor cells in syngeneic C57BL/6 mice has been described (44). Briefly, 8-week-old mice were individually tagged, inoculated subcutaneously in the right flank with 2x 10^5^ MC38 tumor cells and staged to day 7 at which time visible tumors began to be observed. On day 7 post tumor implantation, mice received either BRD509E (5 × 10^3^ CFUs per mouse) or saline as control, both given intraperitoneally. Tumor-bearing mice were intraperitoneally treated with 5 mg/kg (100 μg/dose/mouse) of anti PD-L1 mAb (clone 10F.9G2, BioXCell, USA) or IgG2b isotype control (clone LTF-2, BioXCell) on days 8, 10, 14 and 17 post tumor implantation. Tumor growth was followed by quantitative determination of tumor tissue volume twice a week, measured as the product of the perpendicular diameters using digital calipers, according to the formula: Tumor volume = W^2^/(2 × L). All data of tumor volumes is presented as mean ± SEM.

### 2.3 Bacterial preparation

Viable BRD509E was obtained from a frozen glycerol stock and plated on Tryptone Soy Agar (TSA) supplemented with ampicillin (100 µg/ml) and streptomycin (200 µg/ml). Inoculated TSA plate was incubated overnight at 37°C. On the next day, a single colony from a fresh BRD509E culture was inoculated into 10 ml of Tryptone Soy Broth (TSB) and incubated stationary overnight (16-18 hours) at 37°C. Next, 1 ml of the bacterial culture was added to 9 ml of TSB, creating a dilution factor of 1:10. The freshly prepared *Salmonella* suspension was incubated in an orbital shaker at 37°C, 200 rpm for 2 hours to mid-logarithmic growth phase. To determine the bacterial concentration, optical density was measured at a wavelength of 600 nm using Du-70 spectrophotometer (Beckman coulter Inc. Pasadena, CA, USA). The freshly prepared stock concentration was estimated according to the following formula: OD600 of 0.1 is equivalent to 0.6 × 10^8^ CFU/ml. The final required dose for treatment was prepared by performing appropriate serial dilutions of the bacterial suspension in pyrogen-free PBS.

### 2.4 Bacterial load determination

Tumor-bearing mice were sacrificed following treatment with BRD509E. Tumors, spleens and livers were resected and weighed. Aliquots of tissues were homogenized in cold sterile PBS using Ultra-turrax T-25 tissue homogenizer (Janke & Kunkle, Staufenim Breisgau, Germany) and 1:10 serial dilutions of tissue homogenates were plated on *Salmonella-Shigella* (SS) Agar plates, as previously detailed (26). After overnight incubation at 37°C, bacterial CFUs were counted and tabulated as CFUs per gram of tissue.

### 2.5 Flow cytometry

Spleen and tumor tissues were harvested 21 days post tumor implantation and single-cell suspensions were prepared as previously described (41). Analysis of total spleen and tumor single cells was carried out using a 3-laser 12-color flow cytometry. Washed cells were incubated with FcγR blocking antibody (anti-mouse CD16/32) (Cat# 101302, Biolegend, San Diego, CA) for 30 mins at 4°C and, then, stained with fluorochrome-conjugated primary antibodies- at pre-determined optimum concentrations- for 30 min at 4°C in the dark. The antibodies used in the current study, all purchased from Biolegend, were anti-CD45-APC (Cat# 103112), anti-CD45-PE (Cat# 103106), anti-CD3-BV785 (Cat# 100232), anti-CD4-FITC (Cat# 100509), anti-CD8-APC-Cy7 (Cat# 100714), anti-CD11b-Alexa Flour-488 (Cat# 101217), anti-Ly6G-APC (Cat# 127614), anti-Ly6C-APC/Fire 750 (Cat# 128045), anti-Ly6C-BV785 (Cat# 128041), anti-F4/80-BV421 (Cat# 123137), anti-MHC II (I-A/I-E)-BV785 (Cat# 107645), anti-MHC II (I-A/I-E)-APC-Cy7 (Cat# 107628), anti-CD19-PE (Cat# 115508), anti-PD-L1-PE-Texas Red (Cat# 124324), anti-PD-1-BV605 (Cat# 135220) and anti-LAG-3-BV421 (Cat# 125221). Non-viable cells from spleens and tumors were excluded using Zombie Aqua dye (Biolegend) or 7-AAD viability dye (Biolegend), respectively. Data were collected on 50,000 cells using a BD FACSCelesta flowcytometer (BD biosciences, Mountain View, CA, USA) and analyzed using FlowJo software (BD biosciences).

### 2.6 Immunohistochemistry staining

Formalin-fixed paraffin-embedded tumors (FFPE) were sectioned at a thickness of 5 μm using a rotary microtome (Shandon AS 325, USA) and put on ACP-coated slides. For immunohistochemical staining, deparaffinization, rehydration and endogenous peroxidase activity was performed, per established protocols in our laboratory (45). Antigen unmasking was performed through heat-induced epitope retrieval in which sections were steamed in Tris-EDTA buffer (Sigma Chemicals Co., St Louis, MO) pH 9.0 at 95°C for 10 min. Slides were, then, allowed to cool in the buffer and non-specific binding was blocked by incubating tissue sections with 1% BSA protein block (Sigma). After draining BSA, sections were incubated with a pre-determined concentration of unconjugated, primary monoclonal antibody overnight at 4°C. The primary antibodies used in the current study were as follows: anti-PD-L1 (1/100; ab238697, Abcam, UK), anti-PD-1 (1/1000; ab214421, Abcam), anti-CD4 (1/1000; ab183685, Abcam), anti-CD8 (1/2000; ab209775, Abcam) and anti-granzyme B (1/200; 44153S, Cell Signaling). On the next day, washed tissue sections were incubated with goat polyclonal secondary antibody (HRP polymer) (ab214882, Abcam) for 45 min at room temperature. Following a washing step, DAB chromogen (Dako, Carpinteria, CA) was used as a substrate to detect the activity of HRP; hematoxylin was utilized as a counterstain. All immunohistochemical studies were done using the described protocol except for cleaved caspase-3 staining which was done following the protocol of IHC Detection Kit of the manufacturer (Cell Signaling Technology; #12692). Images were generated with an Olympus BX51 microscope model V-LH100HG (Olympus Corporation, Japan) at x400 magnification.

### 2.7 Quantitative real-time PCR

qRT-PCR was carried out as previously detailed (41). RNA was extracted by TRIzol method and re-purified on Qiagen columns (RNA easy mini kit, Qiagen, Valencia, CA). The quality and quantity of the RNA was determined using the Nanodrop ND-1000 spectrophotometer (Thermo Scientific, Waltham, MA). cDNA was synthesized using Taqman reverse transcription reagents (Applied Biosystems, Foster City, CA) using manufacturer’s protocol. Premade TaqMan primers and probes (Applied Biosystems) were used to study the expression of HPRT (Mm01545399_m1), PD-1 (Mm01285676_m1), mCXCL9 (Mm00434946_m1), mCXCL10 (Mm00445235_m1), IFN-γ (Mm01168134_m1), granzyme B (Mm00442834_m1), CXCL1 (Mm04207460_m1) and CXCL2 (Mm00436450_m1) genes. Transcript levels of target genes were normalized according to the ΔCq method to the respective mRNA levels of the housekeeping gene HPRT. The expression of the target gene is reported as the level of expression relative to HPRT and presented as fold change relative to non-treated mice.

### 2.8 Immunofluorescence staining

Immunofluorescence analysis was used to detect Treg cells in MC38 tumor tissues extracted from mice. The details of the procedure have been described (46). FFPE tissues were sectioned and transferred to gelatin-coated slides. Tissue sections were dewaxed, rehydrated, blocked with 3% BSA and incubated overnight with the primary antibodies: rabbit anti-CD4 (1/1000, ab183685, Abcam) and rat anti-Foxp3 (1/50, 14-5773-82, eBioscience, San Diego, CA, USA). Following a washing step, sections were incubated with anti-rat Alexa Flour-488-labelled (1/200, 712-545-153, Jackson ImmunoResearch, West Grove, PA, USA) and anti-rabbit TRITC-labelled (1/100, 111-025-003, Jackson ImmunoResearch) secondary antibodies for 1 hour in the dark. TO-PRO™-3 iodide (642/661) (T3605, Thermo Fisher Scientific) was used as a counterstain for viable cell nuclei. Last, sections were washed, mounted with fluorescence medium (Dako) and visualized using Nikon C1 laser scanning confocal microscope.

### 2.9 Statistical analysis

Statistical significance between control and treated groups was analyzed using the unpaired, two-tailed Student’s t-test. Survival analysis was performed by Kaplan–Meier survival curves and log-rank test. Tumor doubling time and growth rate constants were determined using multiple linear regression-exponential (Malthusian) growth curves. All analysis was done using the statistical program of GraphPad Prism software version 9 (San Diego, CA). Statistical significance was shown as ^*^ (P < 0.05), ^**^ (P < 0.01), ^***^ (P < 0.001) and ^****^ (P < 0.0001).

### 2.10 Study approval

All studies involving animals were carried out after approval of the animal research ethics committee of the College of Medicine and Health Sciences, UAE University (#ERA_2018_5743).

## 3 Results

### 3.1 PD-1 and its ligand are expressed in MC38 tumor tissues

**Figure 1:**
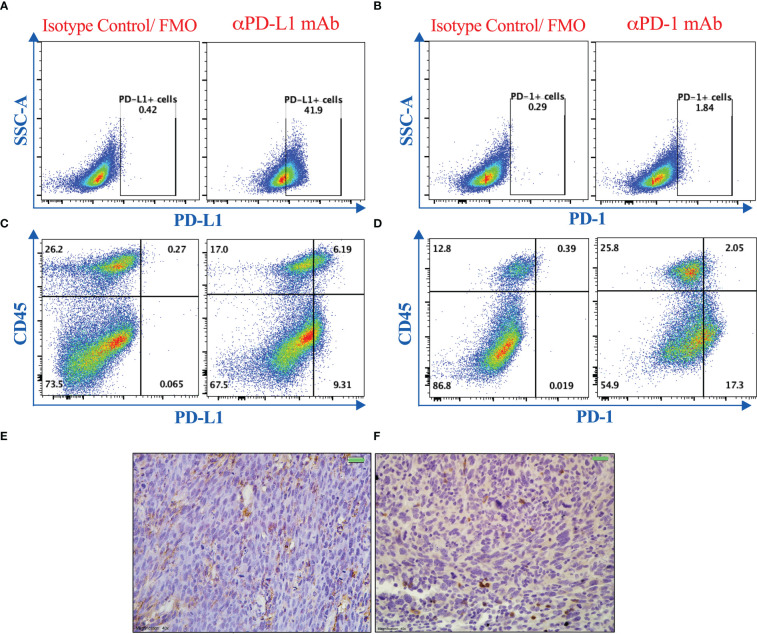
PD-1 and PD-L1 are expressed in MC38 tumors. Representative flow cytometric dot plots showing the expression of PD-L1 **(A, C)** and PD-1 **(B, D)** on MC38 tumor cells grown *in vitro* **(A, B)** or on single cell suspension of dissociated tumor tissues excised from mice 21 post subcutaneous implantation **(C, D)**. Immunohistochemical staining was also used to illustrate the expression of PD-L1 **(E)** and PD-1 **(F)** on MC38 tumor tissue sections. Magnification 400×. Scale bar 20 μm.

Efficient responses to PD-1/PD-L1 blockade treatment using monoclonal antibodies have been associated with adequate expression of these targets within the tumor tissue (47, 48). Therefore, we evaluated the expression of PD-1 and PD-L1 proteins on murine MC38 colon adenocarcinoma cells grown *in vitro* or on tumor tissues that were excised from mice on day 21 post tumor implantation. Using flow cytometric, immunohistochemical and gene expression analyses, we demonstrate that PD-L1 and PD-1 are expressed on MC38 tumor cells (**Figures 1A, B**) as well as on both CD45^+^ and CD45^-^ cells within the tumor tissue (**Figures 1C-F**). PD-1 mRNA expression was also analyzed using RT-PCR on *in vitro*-grown MC38 cells, whole MC38 tumors and normal spleen cells. As shown in **Supplementary Figure 1**, PD-1 expression is upregulated on whole tumor tissue compared to *in vitro*-grown MC38 cells. Nevertheless, it is clear that PD-1 is expressed at higher levels in normal splenic immune cells compared to MC38 cells. The detected expression of PD-1 on tumors is consistent with previous findings (49–51). The confirmed expression of these molecules validated our approach to modulate the immunosuppressive environment through targeting this negative regulatory axis in the MC38 murine colon adenocarcinoma model.

### 3.2 A low dose of attenuated *Salmonella* inhibits MC38 tumor growth *in vivo*

**Figure 2:**
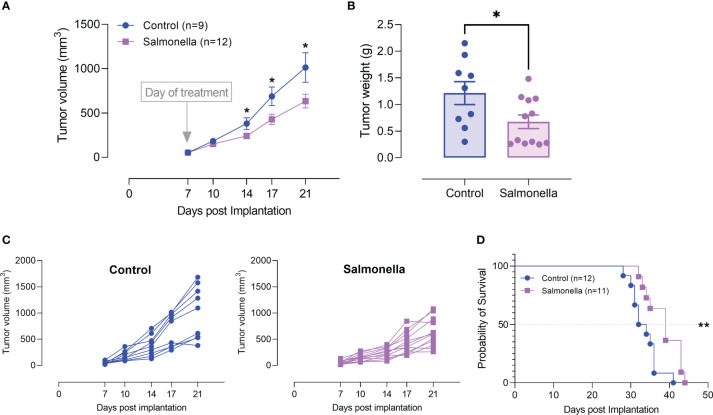
*Salmonella* treatment retards MC38 tumor growth. MC38 tumor cells (2× 10^5^) were subcutaneously implanted into the right flank of C57BL/6 mice. Seven days post implantation, mice were injected intraperitoneally with either *Salmonella* strain BRD509E (~5× 10^3^ CFUs) or saline as control. **(A)** Tumor volumes were measured twice a week for up to 14 days post bacterial treatment. The data is shown as mean ± SEM of 9-12 mice per group, pooled from 2 independent experiments. **(B)** Tumor weights were recorded at the end of the observation period (day 21 post implantation). Each data point represents a single mouse, pooled from two independent experiments. **(C)** Tumor growth curves in each individual mouse in the control and BRD509E-treated groups are presented. **(D)** The effect of BRD509E treatment on the survival of MC38 tumor-bearing mice. Asterisks denote statistically significant differences from control group *(P ≤ 0.05) and ** (P ≤ 0.01).

The potential of attenuated *Salmonella* as an anti-cancer therapeutic agent has been demonstrated in different tumor types. In previous work from our lab, we illustrated the anti-tumor potency of an attenuated strain of *S. typhimurium*, known as BRD509E, against the B16.F1 melanoma model, and its capacity to convert the tumor microenvironment from being immunosuppressive to becoming more immunogenic by modulating the functional properties of myeloid cells (26, 33, 41). The bacterial doses used in the various studies were in the range of 1-5x10^5^ CFUs per mouse. In order to minimize bacteria-associated adverse effects, we wished to test the potential effect of much lower doses of bacteria (2-5-x10^3^ CFUs/mouse), which represent 1/1000 of the LD_50_ dose of the BRD509E strain, when used in combination with other treatments. Using this low dose of bacteria, detection of bacterial colonies in the different organs exhibited wide variability (**Supplementary Figure 2**). Nevertheless, as was generally demonstrated previously (26, 41), preferential homing to tumor tissue was still evident (**Supplemental Figure 2**). The marked variability in the number of colonies detected in mice of the same experimental group could be related to the low bacterial doses used where the bacterial CFUs in the various tissues are reaching the limit of detection. We first evaluated the effect of BRD509E on the growth of MC38 colon adenocarcinoma tumor cells *in vivo*. C57BL/6 mice were implanted with MC38 tumor cells and staged to day 7 at which time visible tumors began to be observed. Tumor-bearing mice were then treated with a single intraperitoneal injection of BRD509E, or saline as control. Significant reduction in tumor growth was observed in mice treated with BRD509E as compared to untreated mice (**Figures 2A-C**). Tumor inhibition was observed as early as 7 days post bacterial treatment (**Figure 2A**) and reached ~40% two weeks after treatment (**Figures 2A, B**). Individual tumor growth curves in control and *Salmonella*-treated mice are shown in **Figure 2C**. In addition to its effect on tumor growth, bacterial treatment resulted in ~18% increase in overall survival (**Figure 2D**).

### 3.3 A low dose of attenuated *Salmonella* enhances the tumor infiltration of CD4^+^ T cells and increases the percentage of MHC II-expressing myeloid cells

**Figure 3:**
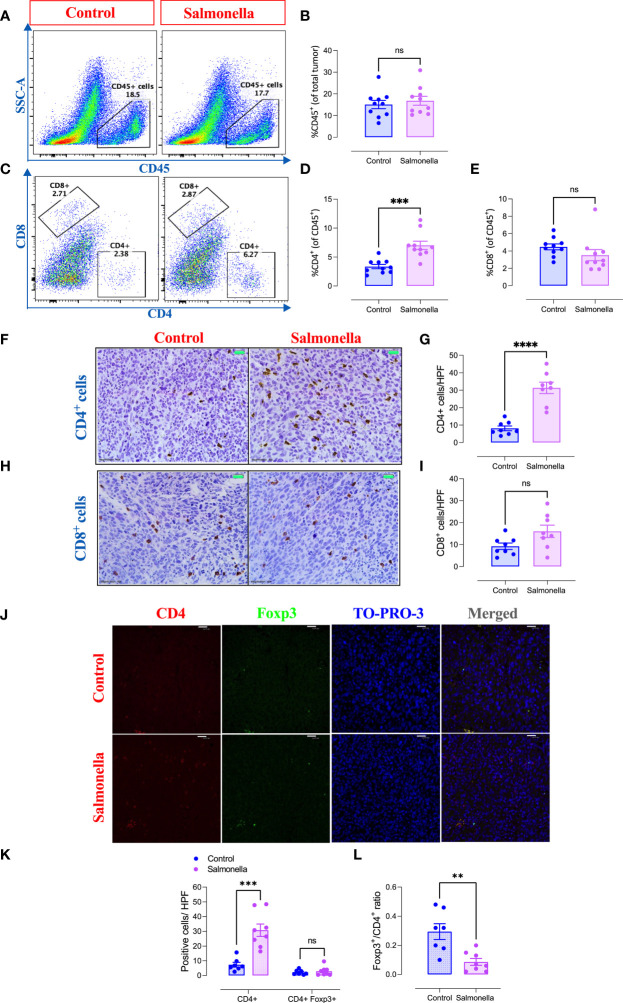
Treatment with *Salmonella* enhances the infiltration of CD4^+^ T cells and decreases the ratio of Tregs/CD4^+^ T cells in MC38 tumors. MC38 tumor-bearing mice were treated with *Salmonella*, or saline as control, on day 7 post tumor implantation. Mice were sacrificed 2 weeks later and tumors were collected for further analysis. The percentages of different intratumoral immune cells were determined using flow cytometry. Representative dot plots and the combined results analyses for the percentages of CD45^+^ immune cells **(A, B)**, CD4^+^ T cells **(C, D)** and CD8^+^ T cells **(C, E)** were shown for each group. Each data point represents a single mouse, pooled from 3 independent experiments. Tumor sections were stained with anti CD4 and anti CD8 antibodies as described in the material and methods section. Representative images and graphs depict the number of CD4^+^ **(F, G)** and CD8^+^ cells **(H, I)**/HPF (high-power field) are presented for each group. Magnification 400×. Each data point represents a single mouse pooled from two independent experiments. Representative immunofluorescent images of CD4, Foxp3, TO-PRO-3 nuclear staining and the merge picture from the control and *Salmonella*-treated groups **(J)**, and the number of CD4^+^ and CD4^+^ Foxp3^+^ cells were quantified/HPF **(K)**. Scale bar 25 μm. The ratio of Tregs (CD4^+^ Foxp3^+^)/CD4^+^ cells were also determined **(L)**. Each data point represents a single mouse pooled from two independent experiments. Asterisks denote statistically significant differences from control group, ** (P ≤ 0.01), *** (P ≤ 0.001), **** (P ≤ 0.0001) and ns (no statistical significance, ≥ 0.05).

**Figure 4:**
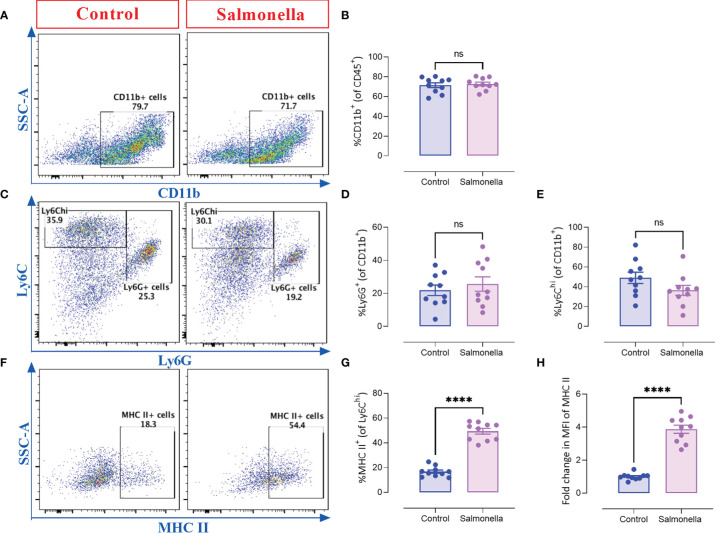
Treating MC38 tumor-bearing mice with *Salmonella* increases the antigen presentation potential of CD11b^+^ Ly6C^hi^ intratumoral myeloid cells. MC38 tumors were collected from control and *Salmonella*-treated mice on day 21 post tumor implantation and the percentages of different intratumoral immune cells were determined using flow cytometry. Representative dot plots and the combined results analyses for the percentages of CD11b^+^ **(A, B)**, Ly6G^+^ **(C, D)**, Ly6C^hi^ **(C, E)** intratumoral myeloid cells and MHC II-expressing cells (gated on CD11b^+^ Ly6C^hi^ cells) **(F, G)** are shown for each group. The expression of MHC II was evaluated using MFI (Median Fluorescence Intensity). **(H)**. Each data point represents a single mouse, pooled from 3 independent experiments. Asterisks denote statistically significant differences from control group, **** (P ≤ 0.0001) and ns (no statistical significance, ≥ 0.05).

Next, we assessed the immunomodulatory effects of BRD509E within the tumor microenvironment by analyzing the changes in the cellular constituents of MC38 tumors using multi-color flow cytometry. The gating strategies used to identify the lymphoid and myeloid subpopulations are illustrated in **Supplementary Figure 3**. The results revealed that although the percentage of total intratumoral CD45^+^ leukocytes in control vs. *Salmonella*-treated groups remained unchanged (**Figures 3A, B**), there was a significant increase (~2.1-fold) in the percentage of CD4^+^ T cells (**Figures 3C, D**) in *Salmonella*-treated tumors. On the other hand, the percentage of tumor infiltrating CD8^+^ T cells was not altered post *Salmonella* treatment (**Figures 3C, E**). The increase in the infiltration of CD4^+^ T cells was also demonstrated morphologically by immunohistochemistry where a 3.8-fold increase was evident in *Salmonella-*treated tumors (**Figures 3F-G**). In contrast, no change in intratumoral CD8^+^ T cells infiltration was observed following treatment with *Salmonella* (**Figures 3H-I**). A more in-depth analysis using immunofluorescence staining revealed that treating tumor-bearing mice with attenuated *Salmonella* decreased the ratio of Tregs/CD4^+^ T cells within the tumor tissue (**Figures 3J-L**). Representative images at lower magnification are presented in **Supplementary Figure 4** for the control and *Salmonella*-treated groups. Interestingly, no significant alteration was observed in the percentage of CD11b^+^ myeloid cells, including Ly6G^+^ and Ly6C^hi^ subpopulations, between the two groups (**Figures 4A-E**). However, a significant increase in the percentage of MHC class II-positive monocytes (CD11b^+^ Ly6C^hi^) was observed in *Salmonella*-treated tumors (**Figures 4F, G**), suggesting an enhancement of the antigen presentation capacity of these cells. Consistent with this observation, an increase in the median expression level of MHC class II antigens was also evident on Ly6C^hi^ myeloid cells (**Figure 4H**). Collectively, these results indicate that a low dose of *Salmonella* could enhance the immunogenicity of MC38 tumors through an increase in the antigen presentation potential of tumor-associated monocytes as well as the extent of CD4^+^ T cell infiltration into the tumor microenvironment.

### 3.4*Salmonella* treatment decreases the percentage of T cells that express inhibitory checkpoint ligands in MC38 tumors

**Figure 5:**
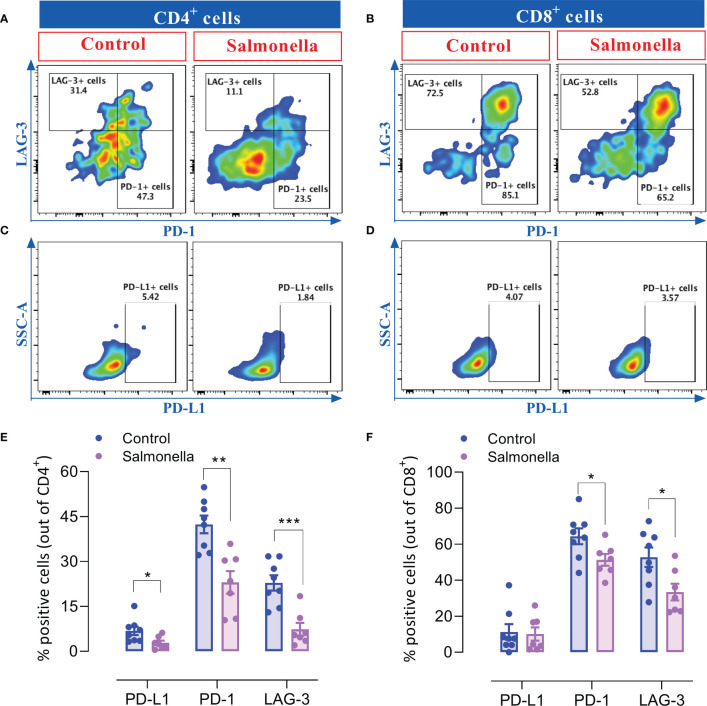
*Salmonella* treatment decreases the percentage of TILs that express inhibitory checkpoint ligands. MC38 tumors were harvested from tumor-bearing mice on day 14 post bacterial treatment and tumor-infiltrating T cells were analyzed for their expression of PD-1, PD-L1 and LAG-3 using flow cytometry. Dot plots representing the percentage of CD4^+^ T cells that express PD-1 and LAG-3 **(A)** or PD-L1 **(C)** are illustrated. Dot plots showing the percentage of CD8^+^ T cells that express PD-1 and LAG-3 **(B)** or PD-L1 **(D)** are also presented. The percentage of CD4^+^ **(E)** and CD8^+^ **(F)** cells that express different inhibitory checkpoint molecules are illustrated. The data is presented using mean ± SEM of 7-8 mice per group pooled from two independent experiments. Asterisks denote statistically significant differences from control group, * (P ≤ 0.05), ** (P ≤ 0.01) and *** (P ≤ 0.001).

Due to the considerable role of immune checkpoint proteins in modulating the tumor microenvironment and determining anti-tumor immune responses, we analyzed the effect of a low dose of *Salmonella* on the expression of inhibitory checkpoint molecules, namely PD-1, PD-L1 and LAG-3, on intratumoral T cells using flow cytometry. Our results indicate that administration of *Salmonella* induced a significant reduction in the percentages of CD4^+^ and CD8^+^ TILs that express PD-1 and LAG-3 checkpoint molecules (**Figures 5A, B** and **E, F**). Flow cytometric analysis revealed that ~42% and 23% of CD4^+^ T cells and ~64% and 53% of CD8^+^ T cells express the PD-1 and LAG-3 inhibitory ligands, respectively, in tumors from untreated mice. Upon treatment with *Salmonella*, these percentages were significantly decreased to an average of ~23% and 7% among CD4^+^ TILs and ~51% and 33% among CD8^+^ TILs. Moreover, a slight decrease in the percentages of PD-L1^+^ cells among CD4^+^ but not CD8^+^ T cells was observed post treatment (**Figures 5C-F**). On the other hand, *Salmonella* treatment did not alter the expression of neither PD-L1 nor PD-1 on tumor CD45-negative cells (**Supplemental Figure 5**). Taken together, the decreased percentage of T cells expressing inhibitory checkpoint ligands within the tumor tissue could potentially contribute to reversing the inhibitory factors exerted on T cells and, thereby, reinvigorating anti-cancer immunity.

### 3.5 *Salmonella* increases the percentage of splenic CD4^+^ T cells and Ly6G^+^ neutrophils in MC38 tumor-bearing mice

**Figure 6:**
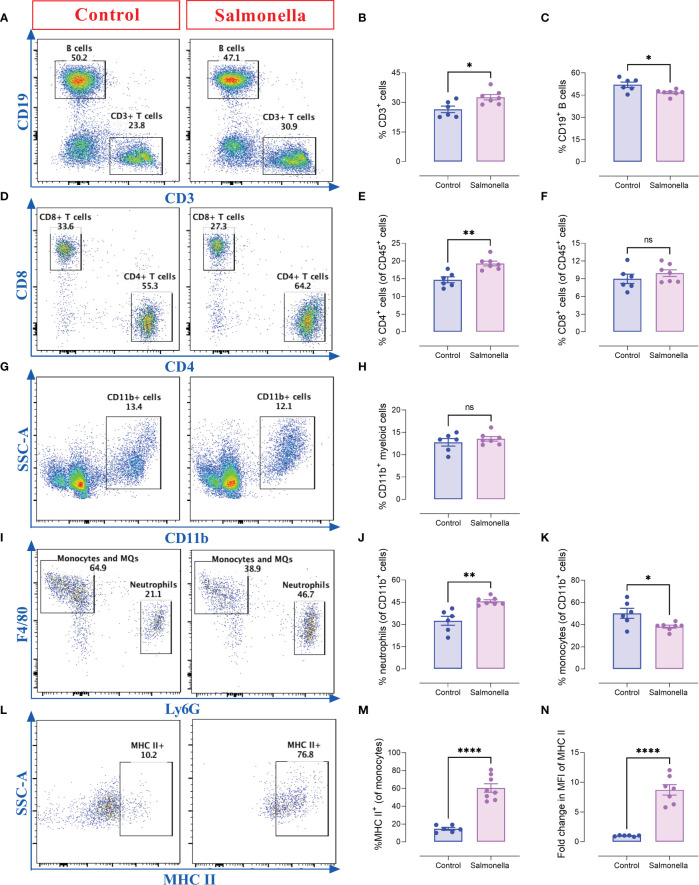
*Salmonella* treatment increases the percentages of splenic CD4^+^ T cells and Ly6G^+^ neutrophils in MC38-tumor bearing mice. Tumor-bearing mice were treated with ~5× 10^3^ CFUs of *Salmonella* on day 7 post tumor implantation. Mice were sacrificed on day 14 post bacterial treatment and spleens were collected for flow cytometric analysis. Representative dot plots and the percentage of T cells **(A, B)**, B cells **(A, C)**, CD4^+^ T cells **(D, E)**, CD8^+^ T cells **(D, F)**, CD11b^+^ myeloid cells **(G, H)**, neutrophils (Ly6G^+^) **(I, J)** and monocytes/macrophages (Ly6G^-^ F4/80^+^) **(I, K)** are illustrated. The percentage of macrophages that express MHC class II was also determined **(L, M)**. The expression of MHC II on monocytes/macrophages was evaluated **(N)**. Each data point represents a single mouse, pooled from two independent experiments. Asterisks denote statistically significant differences from control group, * (P ≤ 0.05), ** (P ≤ 0.01), **** (P ≤ 0.0001) and ns (no statistical significance, ≥ 0.05).

Since the peripheral immune system plays a positive role in anti-tumor therapies, alterations in spleen cell populations were analyzed using flow cytometry. The gating strategies used to identify the lymphoid and myeloid subpopulations are presented in **Supplementary Figure 6**. Our results indicate that a low dose of *Salmonella* induced a slight increase in the percentage of splenic T cells (~27% to ~32%) accompanied with a slight decrease in the percentage of B cells (52% to 47%) (**Figures 6A-C**). The increase in the percentage of T cells was mostly due a significant increase in the percentage of CD4^+^ helper T cells (~15%-19%), but not CD8^+^ cytotoxic T cell population (**Figures 6D-F**). On the other hand, the overall percentage of CD11b^+^ myeloid cells remained unaltered post treatment (**Figures 6G, H**). Regarding the myeloid subpopulation, *Salmonella* resulted in an increase in the percentage of Ly6G^+^ neutrophils from ~32% of splenic myeloid cells to ~46% (**Figures 6I, J**). This was compensated by a decrease in the percentage of Ly6G^-^ F4/80^+^ monocytes/macrophages (**Figures 6I, K**). Nevertheles, F4/80^+^ macrophages showed a significant increase in MHC class II expression (~4.1-fold in % positive cells) following bacterial treatment (**Figures 6L, M**) accompanied with a significant increase in the expression level of MHC class II antigens (**Figure 6N**). In line with these changes, *Salmonella* treatment resulted in ~2.1-fold increase in spleen weights compared to spleens of tumor-bearing mice (**Supplementary Figure 7A**). This was also accompanied with changes in spleen cellularity as demonstrated by ~2.0-fold increase in the absolute spleen cell counts (**Supplementary Figure 7B**). Our results showed that both the lymphoid (**Supplementary Figures 7C-F**) and myeloid (**Supplementary Figures 7G-I**) populations contributed to this increase. Collectively, these results highlight the capacity of a low dose of *Salmonella* to activate systemic immunity through increasing the infiltration of immune cells into the spleen and enhancing the antigen presentation potential of monocytic cells.

### 3.6 A low dose of attenuated *Salmonella* dramatically improves the anti-tumor efficacy of PD-L1 blockade in MC38 colon adenocarcinoma model

**Figure 7:**
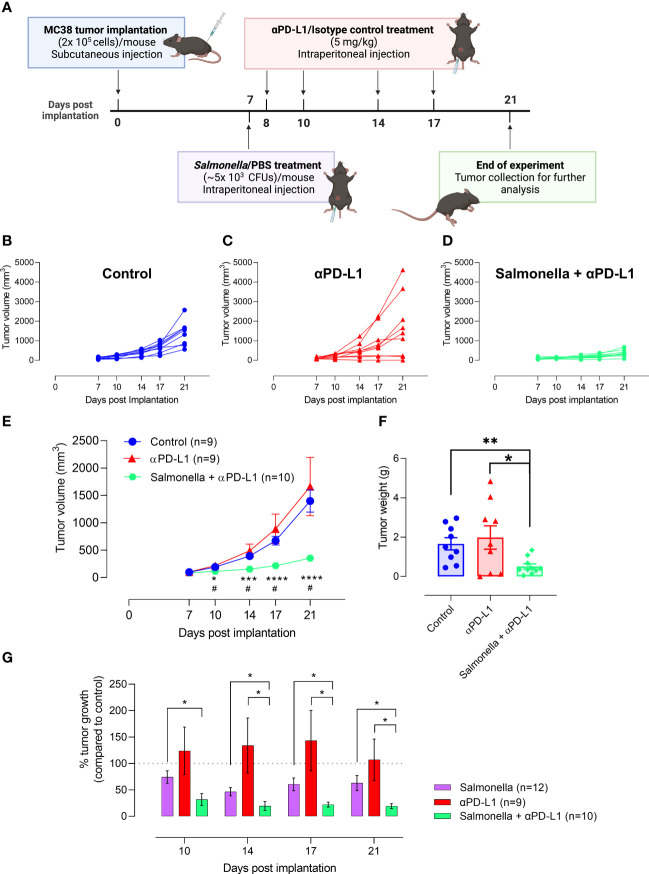
Treatment with *Salmonella* improves the response and anti-tumor efficacy of PD-L1 blockade in MC38 tumors. **(A)** Schematic diagram representing the treatment protocol. MC38 tumor cells were subcutaneously implanted into male C57BL/6 mice. αPD-L1-treated group received 100 μg of αPD-L1 mAb (5 mg/kg) on days 8, 10, 14 and 17 post implantation. Combined αPD-L1 and *Salmonella*-treated group received ~5 × 10^3^ CFUs of BRD509 on day 7 post implantation followed by 4 doses of 5 mg/kg of αPD-L1 mAb on days 8, 10, 14 and 17 post implantation. MC38 tumor growth curve for each mouse in the control **(B)** or αPD-L1-treated **(C)** or αPD-L1 and *Salmonella*-treated **(D)** groups are displayed. The mean tumor volume **(E)** and tumor weights **(F)** are presented for each group. In **(E)**, each data point is presented using mean ± SEM of 9-10 mice per group, pooled from two independent experiments. Each data point in **(F)** represents a single mouse, pooled from 2 independent experiments. **(G)** The percent tumor growth rate in mice treated with *Salmonella* alone, αPD-L1 alone or combination of αPD-L1 and *Salmonella* at different time points. The data is presented using mean ± SEM of 9-12 mice per group. Asterisks denote statistically significant differences between control and combination groups, * (P ≤ 0.05) and ** (P ≤ 0.01). Number signs in **(E)** denote statistically significant differences between control and αPD-L1 groups, # (P ≤ 0.05).

We have demonstrated the potential capacity of a low dose of BRD509E in modulating the immunosuppressive tumor microenvironment through enhancing the intratumoral T cell infiltration, increasing the antigen presentation potential and decreasing the percentage of TILs that express inhibitory checkpoint molecules. Therefore, we next investigated whether BRD509E-induced alterations in the tumor microenvironment are capable to enhance the response and anti-tumor efficacy of PD-L1 blockade immunotherapy against MC38 tumors. We examined the effect of αPD-L1 monotherapy or combination of αPD-L1 and BRD509E on MC38 tumor growth rate. To address this, C57BL/6 mice were inoculated subcutaneously with MC38 tumor cells and staged to day 7 at which time visible tumors began to be observed. In the combination treatment regimen, tumor-bearing mice were treated with a single intraperitoneal injection of BRD509E (5× 10^3^ CFUs/mouse), or saline as control, on day 7 post tumor implantation. Next, αPD-L1 mAb (5 mg/kg) or IgG2b isotype control were intraperitoneally administered to tumor-bearing mice on days 8, 10, 14 and 17 post tumor implantation. The described treatment protocol is illustrated in **Figure 7A**. The results of the current study illustrate that MC38 tumor growth was variable in mice receiving αPD-L1 monotherapy. In our model, only 30% of mice were responsive to αPD-L1 monotherapy treatment and exhibited considerable inhibition in tumor growth (**Figures 7B, C**). On the other hand, combined treatment with αPD-L1 and BRD509E resulted in effective inhibition of MC38 tumor growth in 100% of mice (**Figures 7B, D**). Overall, the combination treatment significantly inhibited MC38 tumor growth compared to control, achieving ~75% inhibition in tumor volume by day 21 post tumor implantation (**Figure 7E**). This is consistent with the significant reduction in the weights of tumors which were determined at the end of the observation period (**Figure 7F**). Moreover, it is worth noting that tumor regression in mice receiving αPD-L1 and *Salmonella* was observed as early as day 3 post bacterial treatment (**Figure 7E**).

In comparison to *Salmonella* treatment, the tumor growth rate in αPD-L1 and *Salmonella*-treated mice was significantly lower than that observed with *Salmonella* alone throughout the observation period (**Figure 7G**). On day 21 post tumor implantation, MC38 tumor growth in *Salmonella*-treated mice was ~60% compared to control, whereas tumor growth in combination-treated mice was ~20% of its growth in the control group (**Figure 7G**). This superior effect of combination treatment in retarding tumor growth compared to *Salmonella* monotherapy was also observed as early as 3 days post bacterial treatment. To further validate the differences in the experimental groups, we calculated the tumor doubling time and the growth rate constant for each experimental group (**Supplemental Table I**). The findings show that there was no difference in tumor growth characteristics in mice treated with saline or isotype control antibody. Moreover, mice treated with α-PD-L1 antibody showed overall tumor growth that was close to control mice (a mere 4% increase in tumor doubling time). This fact is perhaps a reflection of the variability of the responses in this experimental group. Treatment with a low dose of Salmonella resulted in slower tumor growth with doubling time increasing from a mean of 3.78 in control mice to 4.13 days in Salmonella-treated mice (an increase of 9% in doubling time over control). Finally, mice subjected to combination treatment exhibited the most pronounced retardation in tumor growth with doubling time increasing from 3.8 to 6.36 days, an increase of 67% compared to control (and 59% increase compared to Salmonella alone group). Taken together, these results collectively indicate that (1) a low dose of *Salmonella* is successful in improving the response and enhancing the anti-tumor efficacy of PD-L1 blockade in MC38 tumor model and (2) the combined treatment of *Salmonella* and PD-L1 blockade improves tumor inhibition more efficiently than monotherapy.

### 3.7 Combined treatment of αPD-L1 and *Salmonella* is associated with a significant increase in the infiltration of CD45^+^ immune cells into MC38 tumors

**Figure 8:**
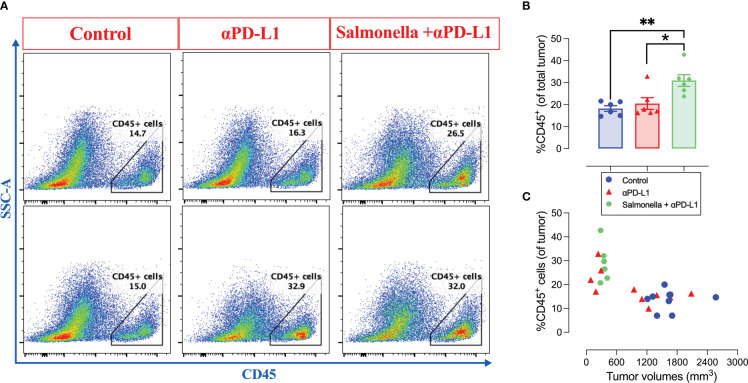
The combination of αPD-L1 and *Salmonella* enhances the infiltration of CD45^+^ immune cells into MC38 tumors. MC38 tumor-bearing mice were treated with αPD-L1 or combination of *Salmonella* and αPD-L1 or isotype control. On day 21 post tumor implantation, mice were sacrificed, tumors were collected and the percentage of CD45^+^ cells were determined using flow cytometry. **(A)** Representative dot plots showing the percentage of CD45^+^ cells out of MC38 tumors in control, αPD-L1 and combination-treated groups. **(B)** Quantification of the percentage of CD45^+^ immune cells in the three groups. The correlation between tumor volumes and the infiltration of CD45^+^ cells is illustrated **(C)**. Each data point in **(B)** represents a single mouse, pooled from 2 independent experiments. Each data point in **(C)** represents a single mouse, pooled from 3 independent experiments for control and αPD-L1-treated groups and from 2 independent experiments for combination-treated group. Asterisks denote statistically significant differences from control group, * (P ≤ 0.05) and ** (P ≤ 0.01).

Next, we aimed to study the effect of combined treatment on the immune components of MC38 tumor microenvironment. To address this, tumors were collected from control, αPD-L1-treated and combination-treated mice on day 21 post implantation and intratumoral immune cells were analyzed using flow cytometry. Our results show that the combined treatment with αPD-L1 and *Salmonella* led to a significant 1.7-fold increase in the infiltration of CD45^+^ immune cells into the tumor site compared to the control group (~18% vs. 31%) (**Figures 8A, B**). This significant increase in the percentage of CD45^+^ immune cells was not observed following treatment with *Salmonella* alone (**Figure 4A**). Regarding PD-L1 blockade, the percentage of CD45^+^ cells in tumors from mice treated with αPD-L1 were variable depending on the individual mouse response to the treatment (**Figures 8A-C**). As clearly illustrated in **Figure 8C**, there was a direct correlation between the treatment outcome and extent of tumor infiltration of CD45^+^ immune cells among αPD-L1 and combination-treated groups compared to controls, effectively demonstrating that the beneficial responses were observed in tumors harboring a high fraction of CD45^+^ cells.

### 3.8 Combination treatment enhances the infiltration of TILs and upregulates the expression of anti-tumor effector molecules

**Figure 9:**
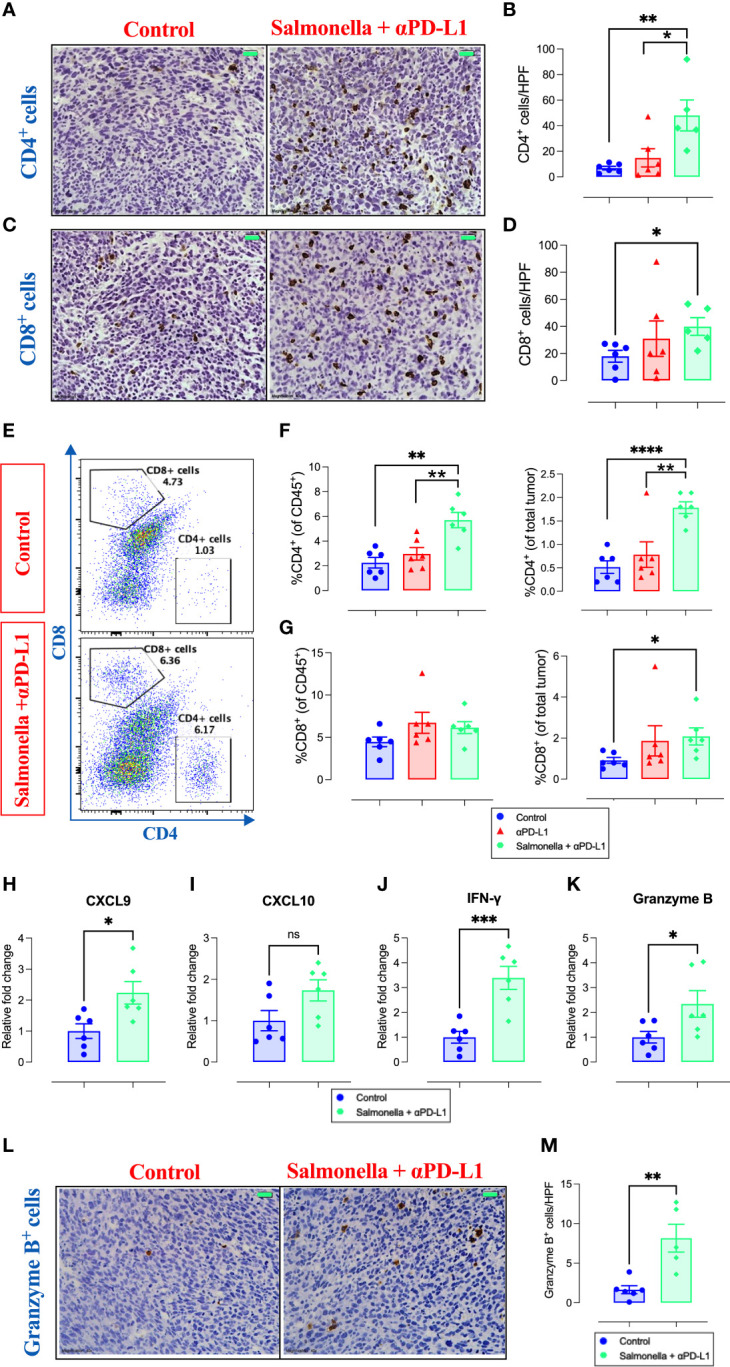
Combination treatment increases MC38 tumor infiltration by CD4^+^ and CD8^+^ T cells and the expression of their effector molecules. MC38 tumor tissues were resected from non-treated, αPD-L1 and combination-treated mice on day 21 post implantation for further analyses using immunohistochemistry, flow cytometry and qRT-PCR. Representative immunohistochemical images for CD4 **(A)** and CD8 **(C)** in tumor tissues are presented for the control and combination-treated groups. Magnification 400×. Scale bar 20 μm. CD4^+^ **(B)** and CD8^+^ **(D)** cells were quantified in 15 HPF for control, αPD-L1 and combination-treated groups. Each data point represents the average of positive cells/HPF from a single mouse, pooled from 2 independent experiments. Representative flow cytometric dot plots and the combined result analyses for the percentages of CD4^+^ **(E, F)** and CD8^+^ **(E, G)** cells in MC38 tumors are illustrated. RNA was extracted from total MC38 tumor tissues and gene expression levels were determined using qRT-PCR. The effect of combination treatment on the expression levels of CXCL9 **(H)**, CXCL10 **(I)**, IFN-γ **(J)** and granzyme B **(K)** was assessed. Representative images for granzyme B staining in tumor tissues are presented for each group **(L)**. Graph depicts the number of cells/HPF **(M)**. Magnification 400×. Scale bar 20 μm. Each data point represents a single mouse pooled from two independent experiments. Asterisks denote statistically significant differences from control group, * (P ≤ 0.05), ** (P ≤ 0.01), *** (P ≤ 0.001) and **** (P ≤ 0.0001).

Next, we examined the effect of combination treatment on the different intratumoral immune cell subpopulations using immunohistochemistry and flow cytometry. Immunohistochemical staining revealed that combination treatment with *Salmonella* and αPD-L1 significantly increased the infiltration of both CD4^+^ (**Figures 9A and B**) and CD8^+^ T cells (**Figures 9C and D**) into MC38 tumor site. It worth noting that the magnitude of increase among CD4^+^ cells was much greater than that observed among CD8^+^ cells (~7-fold versus ~2-fold). Flow cytometric analysis revealed that combination treatment resulted in an increase in the % of CD4^+^ T cells (~2.5-fold and ~1.9-fold) compared to the control or αPD-L1-treated groups, respectively (**Figures 9E, F**). The percentage of CD8^+^ T cells out of CD45^+^ cells remained unchanged following combination treatment (**Figures 9E, G**). The increased infiltration of CD8^+^ TILs cells into tumor tissues (as observed in immunohistochemical staining) is attributed to the increased infiltration of CD45^+^ immune cells that include similar percentages of CD8^+^ T cells in the control and combination-treated groups and this is demonstrated as an increase in the percentage of these cells out of total tumor tissue (**Figure 9G**). Based on gene expression analysis that was performed on total tumor tissues using qRT-PCR, we demonstrated that the enhanced recruitment of T cells could be attributed to the increased expression of related chemokines where a 2.2-fold increase in the expression of CXCL9 (**Figure 9H**) and a trend of increase in CXCL10 (**Figure 9I**) were evident. In order to establish the link between the significant inhibition in tumor growth and the increased infiltration of T cells observed in combination-treated tumors, we also utilized qRT-PCR to examine the expression of anti-tumor effector agents (IFN-γ and granzyme B) that are known to be produced and secreted by T cells. The results demonstrate that the enhanced anti-tumor immune response in combination-treated group was accompanied by a pronounced upregulation in the expression of IFN-γ (~3.9-fold) and cytotoxic granzyme B (~2.3-fold) at the level of total tumor tissue (**Figures 9J, K**). Consistent with these findings, we also found increased number of granzyme B-secreting cells in MC38 tumors following treatment with the combination of *Salmonella* and αPD-L1 using immunohistochemistry (**Figures 9L, M**).

### 3.9 Combined treatment is associated with a reduction in intratumoral Ly6G^+^ myeloid cells and a significant increase in Ly6C^hi^/Ly6G^+^ ratio in MC38 tumors

**Figure 10:**
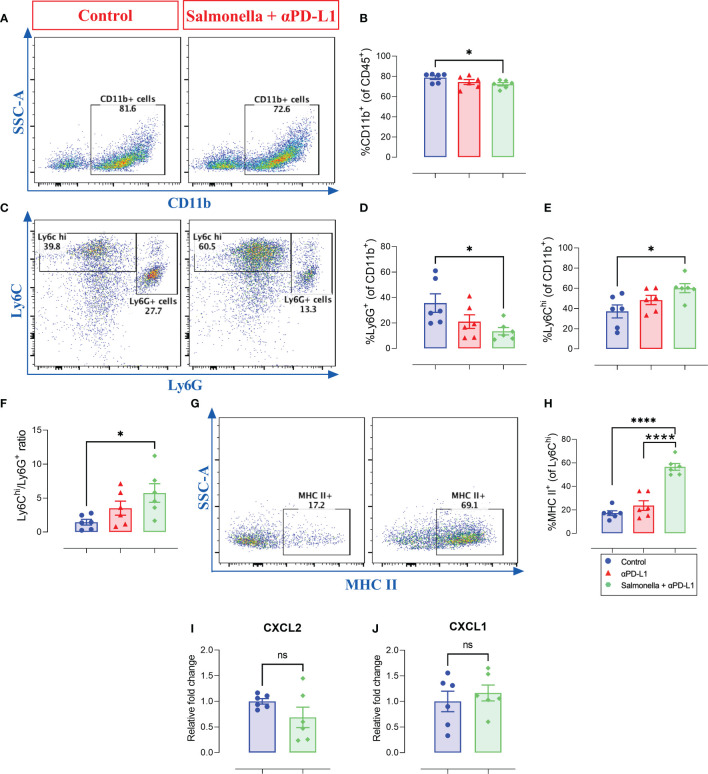
Combined treatment alters the intratumoral myeloid cells compartment in MC38 tumor-bearing mice. MC38 tumor tissues were resected from non-treated, αPD-L1 and combination-treated mice on day 21 post implantation for flow cytometric analysis. Representative dot plots and the combined result analyses for the percentages of CD11b^+^ myeloid cells **(A, B)**, Ly6G^+^ **(C, D)**, Ly6G^-^ Ly6C^hi^ **(C, E)** cells in MC38 tumors are illustrated. The expression of MHC II on Ly6C^hi^ cells was also assessed **(G, H)**. The levels of CXCL2 **(I)** and CXCL1 **(J)** expression in total MC38 tumor tissues were determined using qRT-PCR. Each data point represents a single mouse pooled from two independent experiments. Asterisks denote statistically significant differences from control group, * (P ≤ 0.05), **** (P ≤ 0.0001) and ns (no statistical significance, ≥ 0.05).

Analysis of the intratumoral CD11b^+^ myeloid population revealed a small but significant decrease in overall percentage following treatment with *Salmonella* and αPD-L1 compared to controls (**Figures 10A, B**). However, the composition of this myeloid population was profoundly altered following treatment through a decrease in the percentage of Ly6G^+^ cells (~62% reduction) and a corresponding increase in the percentage of Ly6C^hi^ cells (~62% increase) (**Figures 10C-E**). In other words, the improved anti-tumor effects of combination treatment were shown to be associated with increased Ly6C^hi^/Ly6G^+^ ratio (**Figure 10F**) in comparison to untreated group (~1.4 vs. ~5.7). This finding was not observed among *Salmonella*-treated group. Moreover, combined treatment with *Salmonella* and αPD-L1 significantly increased the percentage of CD11b^+^ Ly6C^hi^ monocytes that express MHC class II in comparison to the control and αPD-L1-treated groups (**Figures 10G, H**), suggesting an increase in the antigen-presentation potential of these cells. The observed reduction in the percentage of Ly6G^+^ cells correlated with a decrease in the expression of CXCL2 (**Figure 10I**), a neutrophil chemotactic factor, but not CXCL1 (**Figure 10J**). In addition to the enhanced tumor infiltration by T cells, these results indicate that combination treatment with *Salmonella* and αPD-L1 modulated the MC38 tumor microenvironment and increased its immunogenicity in favor of tumor inhibition.

### 3.10 Combination treatment with *Salmonella* and αPD-L1 increases tumor apoptosis

**Figure 11:**
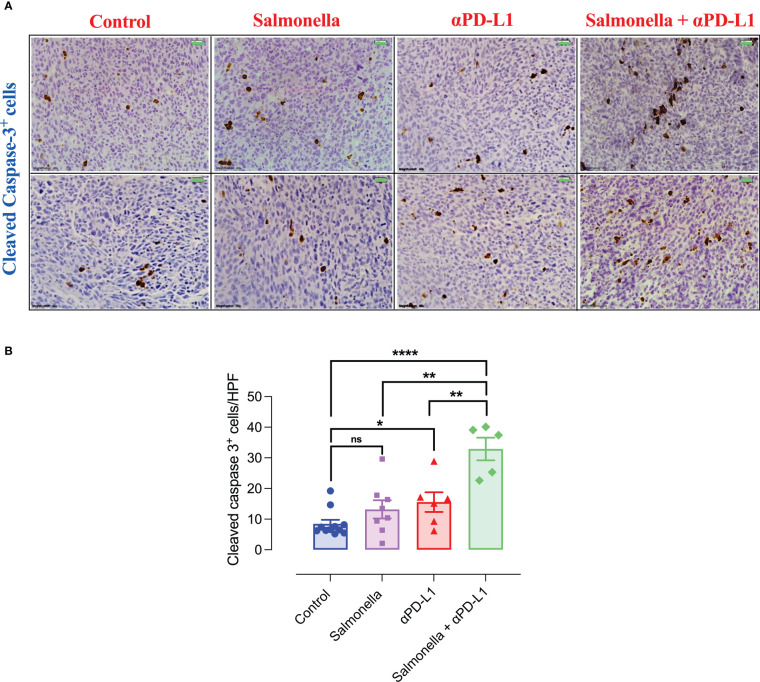
Combination treatment of *Salmonella* and αPD-L1 induces apoptosis more efficiently than monotherapy. Representative immunohistochemical images for cleaved caspase-3 in tumor tissues are presented for control, *Salmonella*, αPD-L1 and combination-treated groups **(A)**. Magnification 400×. Scale bar 20 μm. Cleaved caspase 3^+^ cells were quantified in 15 HPF for the different groups **(B)**. Each data point represents the average of positive cells from a single mouse, pooled from 2 independent experiments for all groups except for the control group, from three independent experiments. Asterisks denote statistically significant differences from control group, * (P ≤ 0.05), ** (P ≤ 0.01), *** (P ≤ 0.001) and ns (no statistical significance, ≥ 0.05).

To further characterize the mechanisms underlying the enhanced anti-tumor activities of combination therapy, we determined the effect of *Salmonella* and αPD-L1 on tumor apoptosis through performing immunohistochemical staining for activated caspase 3, which is one of the key mediators of apoptosis. The levels of cleaved caspase 3 were quantified following treatment with either *Salmonella* or αPD-L1 alone or combination of both. Our results demonstrated that treatment with either *Salmonella* or αPD-L1 induced a 1.6-fold or 1.8-fold increase in the number of cells undergoing apoptosis, respectively (**Figures 11A, B**). In contrast, combination treatment resulted in the largest increase (3.9-fold) in the level of cleaved caspase-3 among all the treated groups (**Figures 11A, B**). In other words, combined therapy succeeded in promoting apoptosis more efficiently than monotherapy.

## 4 Discussion

Immune checkpoint inhibitors have achieved unprecedented success in cancer immunotherapy and have become one of the principal modalities for cancer treatment. ICIs that target PD-1/PD-L1 axis, in particular, have been utilized to treat different types of cancer, however; the response rate among treated patients remains rather modest with CRC being at the lower of the spectrum (52, 53). In the past decade, efforts have been made to overcome resistance and enhance the therapeutic outcome of the treatment through combining PD-1/PD-L1 inhibitors with other therapies (23). In the current study, a low dose of attenuated *S. typhimurium* (~5x 10^3^ CFUs) was utilized to ameliorate the immunosuppressive tumor microenvironment and, therefore, improve the response rate and therapeutic efficacy of PD-L1 blockade in the murine MC38 colon adenocarcinoma model. Different studies showed variability in the response of this model to αPD-L1 mAb. While some studies demonstrated that αPD-L1 immunotherapy inhibited tumor growth by ~60-70% (54–56) others showed that only ~20-30% of tumor-bearing mice responded efficiently to the treatment with ICIs (57, 58). It is important to highlight that the *S. typhimurium* strain used in this study (BRD509E) is an avirulent, double auxotrophic, strain which has been extensively used to vaccinate animals against virulent *Salmonella* (42, 43). The LD_50_ of BRD509E in C57BL/6 mice is >2x10^6^ CFUs when given systemically. Thus, there is no appreciable toxicity associated with this strain when given at the low doses used in our study.

Our results indicate that, even at this low dose, attenuated *Salmonella* organisms were able to effect significant changes in the MC38 tumor microenvironment in favor of tumor inhibition through enhancing the infiltration of CD4^+^ T cells and increasing the antigen presentation capacity of intratumoral macrophages through upregulating the expression of MHC class II. It is reasonable to conclude that *Salmonella*-induced alterations at the tumor site helped to overcome the limitations of the treatment with ICIs. It has been proposed that a favorable response to PD-1/PD-L1 inhibitors is dependent on the extent of T cell infiltration into the tumor tissue (59, 60). Tumors with poor immunogenicity were shown to be unresponsive to PD-1/PD-L1 blockade due to insufficient pre-existing T cells at the tumor site (61, 62). In addition, lack of T cell recognition of tumor antigens is considered a barrier to the success of ICIs that rely on neoantigen presentation and T-cell priming (63). In our point of view, the illustrated capacity of *Salmonella* to broaden the response to PD-L1 inhibitors is, in part, mediated by its ability to increase the infiltration of intratumoral T cells and its potential to induce direct tumor killing and release tumor-associated antigens leading to the induction of antigen-presenting cells, antigen cross-presentation and activation of T cells. The observed increase in the percentage of myeloid cells that express MHC class II proteins would most likely promote the activation of helper CD4^+^ T cells.

Another important finding of this study relates to the ability of a single intraperitoneal injection of attenuated *Salmonella* to induce a significant decrease in the percentage of CD4^+^ and CD8^+^ TILs that express the inhibitory immune checkpoint molecules PD-1 and LAG-3, suggesting its potential to reverse tumor immune tolerance and diminish T-cell exhaustion and, therefore, enhance T cell-mediated anti-tumor immunity. In this context, it is worth highlighting that targeting a single immune checkpoint molecule with ICIs enhances the upregulation of alternate molecules including PD-1, CTLA-4, TIM-3 and LAG-3 (64–66). This phenomenon could underlie the low response rate among treated patients in addition to relapses due to secondary resistance. Based on our observation, *Salmonella* could interfere with this compensatory upregulation, break T cell dysfunction and act as a co-target therapy. The underlying mechanisms for the downregulation of the expression of inhibitory checkpoints are not sufficiently investigated. One study correlated *Salmonella*-induced downregulation of PD-L1 in tumor tissues to the inhibition of AKT/mTOR/p70S6K signaling pathway post treatment with *Salmonella* in a murine tumor model (67). Moreover, the inhibition of immune checkpoints expression could also be delivered through *Salmonella*’s capacity to downregulate the expression of certain immunosuppressive factors including TGF-β that induces the expression of PD-1 on T cells (41, 68). It is important to observe that the expression of PD-1 and PD-L1 can be also regulated at the posttranslational level by glycosylation, fucosylation and ubiquitination reactions (69). In this context, it is noteworthy that we have detected an upregulation in PD-1 cell surface expression on MC38 tumor tissue. This upregulation could result from enhanced gene transcription, as shown in the results of the RT-PCR analysis, as well as posttranslation modification of the PD-1 protein by glycosylation and fucosylation in the ER and Golgi apparatus (70). The significance of tumor-specific PD-1 expression on the observed enhancement with the combination treatment is under investigation.

Tumor-targeting immunotherapies require the systemic immune responses to be effective. Herein, the systemic administration of a low dose of attenuated *Salmonella* resulted in a modest 2-fold increase in spleen weights. This level of splenomegaly is significantly lower than the 5- to 10-fold increase in spleen weights seen when higher doses (10^5^-10^6^ CFUs per mouse) of BRD509E were used (43). Several lines of evidence documented that splenomegaly associated with *Salmonella* infections is mainly due the initiation of extramedullary erythropoiesis and prominent increase in the immature erythroid compartment (71, 72). Moreover, it is worth noting that *Salmonella* organisms replicate extensively within phagocytes of spleen, liver and bone marrow in systemic infections (39, 73). In order to eliminate the bacteria, chemokine-dependent recruitment of innate immune cells, including neutrophils, to the infected site is initiated (73, 74). This is followed by a marked activation and expansion of CD4^+^ and CD8^+^ T cells that play an essential role in controlling bacterial infection (75–77). The current study illustrates that treatment using exceedingly small doses of *Salmonella* organisms is associated with an attenuated and transient level of splenomegaly, which is evidence of mobilizing systemic immunity. However, these responses would not be expected to result in any long-term adverse effects in the host (43, 78).

Given the ability of a low dose of *Salmonella* to restructure the immune component of tumor microenvironment and overcome the major limitations to achieve favorable therapeutic outcome with ICIs, we utilized *Salmonella* in combination with αPD-L1 to treat MC38 tumor-bearing mice. Monotherapy with αPD-L1 resulted in variable response rates among treated mice and this could be associated with the frequency and proliferation of Tregs in tumor tissues and multiple immune organs (79). In our settings, ~30% of mice were responsive to αPD-L1 while others were comparable to the control group. On the other hand, the combination of αPD-L1 and *Salmonella* remarkably improved the response rate and therapeutic efficacy of the treatment in all animals. Overall, the combination inhibited MC38 tumor growth more efficiently than monotherapy throughout the entire experimental period and this has been associated with changes in the intratumoral immune system compartment. Flow cytometric analysis demonstrated a correlation between the anti-tumor potency of the treatment and the percentage of CD45^+^ immune cells at the tumor site, in which tumors from combination-treated mice were present with a higher fraction of CD45^+^ cells compared to untreated mice (~70% increase). The percentages of CD45^+^ cells in tumors from αPD-L1-treated were variable depending on the individual mouse response to the treatment. It is noteworthy that *Salmonella* treatment alone did not alter the fraction of CD45^+^ cells indicating that the observed increase in the infiltration of immune cells is specific to the improved anti-tumor effect of combination treatment.

A more in-depth analysis revealed that the combination treatment with *Salmonella* and αPD-L1 enhanced the intratumoral infiltration of both CD4^+^ and CD8^+^ cells in comparison to either treatment alone. Unexpectedly, the combined treatment largely induced a CD4^+^ T cell response that is more prominent than CD8^+^ T cell response in MC38 tumors. This preponderance of CD4^+^ T cell responses over that of CD8^+^ T cell could be attributed to the relatively larger spectrum of MHC class II-restricted epitopes due to the more relaxed binding requirements compared to MHC class I-restricted epitopes (80). Evidence claimed that efficient responses to ICIs require CD4^+^ T cell responses to MHC class II-restricted antigens (81). The increased understanding of the role of CD4^+^ T cells as an integral part of anti-tumor immune response allowed us to explain the significant increase in the infiltration of CD4^+^ cells along with the improved anti-tumor potential of combination treatment. Several studies documented the anti-tumor potency of CD4^+^ T cells and their role in directing and sustaining efficient anti-tumor immune responses. The activation and polarization of CD4^+^ T cells into Th1 phenotype is accompanied by a heightened ability to produce and secrete effector cytokines such as IFN-γ and TNF-α that have direct anti-tumor activities (82). In a similar manner to CD8^+^ T cells, CD4^+^ T cells were shown to induce direct cytotoxicity against tumor cells in pre-clinical tumor models (83–85). Besides, CD4^+^ T cells play an essential role in supporting the effector function of CD8^+^ cytotoxic T cells through (1) secreting IL-2 which promote the activation and proliferation of CD8^+^ T cells (86), and (2) activating and maintaining the proinflammatory cross presenting DCs through the engagement of CD40L on activated CD4^+^ T cells with CD40 on DCs (87, 88). This interaction upregulates the expression of the co-stimulatory molecules CD80 and CD86 on DCs and promotes their secretion of IL-12. This, in turn, aids in providing the required signal for efficient CTL priming. Besides the increased fraction of T cells in MC38 tumors post combination treatment, our data suggest an increase in anti-tumor potential of these cells as assessed by the levels of IFN-γ and granzyme B. IFN-γ is predominantly secreted by NK cells, NKT cells, CD8^+^ CTLs and Th1 CD4^+^ cells, and plays an essential role in modulating the different immune cells in favor of tumor growth inhibition (89). Moreover, CTLs exert their anti-tumor effects through the introduction of granzyme B to the cytosol of target tumor cells and, therefore, induce rapid cell death (90).

In addition to alterations in T cells, our study also revealed a connection between the responsiveness to combination treatment and the intratumoral myeloid populations. Although the overall percentage of intratumoral CD11b^+^ myeloid cells was not altered substantially, the treatment resulted in a marked decrease in the percentage of Ly6G^+^ granulocytic cells and a corresponding increase in the percentage of Ly6G^-^ Ly6C^hi^ monocytic population. In addition, the combination treatment led to enhanced expression of MHC class II- induced by IFN-γ- on Ly6C^hi^ macrophages, suggesting an improvement in the antigen-presentation potential of these cells. This is consistent with the increased frequency of intratumoral CD4^+^ T cells observed in these mice. Based on existing evidence, the abundant intratumoral myeloid compartment is mainly composed of TAMs, MDSCs, DCs and tumor-associated neutrophils that exhibit divergent functions molded by the local and systemic environmental stimuli during tumor development (91–93). Most myeloid cells in the tumor microenvironment promote survival, proliferation and migration of cancer cells through either direct cell-cell contact or/and release of soluble factors, in addition to their ability to stimulate tumor angiogenesis, induce immune suppression and promote drug resistance (94). On the other hand, intratumoral myeloid cells could display potent anti-tumor properties and exert tumoricidal effects. In our model, the enhanced anti-tumor effect of combination treatment correlated with a remarkable decrease in the percentage of Ly6G^+^ neutrophils suggesting that these cells are associated with PMN-MDSCs that play considerable pro-tumorigenic and immunosuppressive roles (95). The inverse relationship between beneficial anti-tumor effects and intratumoral PMN-MDSCs has been described in other studies (96–98). In addition, combination treatment led to an increase in the percentage of Ly6C^hi^ macrophages, which are known to exhibit considerable phenotypic and functional heterogeneity and could contribute to either pro- or anti-tumor immunity (99). The exact role of these cells in the context of combination treatment of MC38 tumors requires further investigation, particularly in terms of conducting cellular assays that would help us to determine the function of these cells in MC38 tumors. In the context of our findings and given the known plasticity of intratumoral myeloid cells (93), it is reasonable to assume that the combination treatment with *Salmonella* and αPD-L1 could induce transcriptional changes through altering the expression of genes involved in chemotaxis, metabolism, and differentiation, effectively reprograming the function of Ly6C^hi^ cells toward being anti-tumor effector cells (100). Long et al. has linked the anti-fibrotic potential of anti-CD40 treatment to the recruitment of Ly6C^hi^ monocytes in pancreatic cancer (101). The immunosuppressive tumor microenvironment, including MDSCs, is considered a major limitation to the success of ICIs in CRC (102). Therefore, the ability of *Salmonella* and αPD-L1 combination to restructure the myeloid compartment of MC38 tumor model in favor of tumor inhibition provides a novel therapeutic approach to treat CRC.

In support of our findings, a similar observation of the improved anti-tumor effect has been documented with the combination of IDO-targeting *Salmonella* and PD-1/CTLA-4 inhibitors in the murine LLC1 tumor model in comparison to either *Salmonella* alone or PD-1/CTL1-4 mAb alone (103). Using subtherapeutic doses of IDO-targeting *Salmonella*, they observed an increase in the antigen presentation machinery and costimulatory molecules in intratumoral myeloid cells, decrease in the frequency of Tregs in LLC1 tumor microenvironment and inhibition in the expression of inhibitory immune checkpoint ligands on splenic immune cells. On the other hand, the frequency of TILs remained unchanged post treatment with *Salmonella*. *Salmonella*-induced alterations in the expression of inhibitory checkpoint molecules was only investigated among splenic immune cells but not on intratumoral immune cells. In parallel with our study, the enhanced anti-tumor effect in combination treatment was mediated by the increased infiltration of CD45^+^ immune cells, CD4^+^ and CD8^+^ TILs in addition to decreased percentage of CD11b^+^ Ly6G^+^ cells. Contrary to our findings, the improved anti-tumor efficacy in their settings was associated with a decrease in the percentage of CD11b^+^ Ly6C^+^ intratumoral immune cells. Moreover, the enhanced anti-tumor effect of combination treatment was correlated with a decrease in the frequency of Tregs compared to treatment with PD-1/CTLA-4 inhibitors. Despite these changes, they illustrated that TILs-associated activation and effector markers including IFN-γ, granzyme B and CD62L remained unchanged post treatment with *Salmonella* and PD-1/CTLA-4 inhibitors. Another study illustrated enhanced rejection of B16-OVA melanoma cells using ovalbumin-producing *Salmonella* and αPD-L1 compared to *Salmonella* alone or combination of PD-L1 and CTLA-4 inhibitors through enhancing the expansion of CD8^+^ T cells (104). The same study documented that *Salmonella* is incapable to enhance the therapeutic efficacy of CTLA-4 inhibitors. Others reported that combined treatment with IDO-targeting *Salmonella* and αPD-1 inhibited the growth of CT26 or MC38 tumors more efficiently than αPD-1 treatment alone, however; no additional anti-tumor effects of combination treatment has been observed in comparison to *Salmonella* alone (105).

The current study elucidated insights into the mechanism underlying the observed alterations in the immune cell components in MC38 tumors post combination treatment as shown by a significant increase in the expression level of CXCL9 and a trend of increase in CXCL10. Both CXCL9 and CXCL10 are chemokines secreted by monocytes, endothelial cells, fibroblasts and cancer cells in response to stimulation by IFN-γ (106). These chemokines function through paracrine signaling and regulate the recruitment of immune cells including CTL, NK cells, NKT cells and M1 macrophages, and promote Th1 polarization and activation (106–108). Our results also suggested that combination treatment decreased the expression level of CXCL2. Several lines of evidence highlighted the critical role of CXCL2 in recruiting tumor-associated neutrophils and promoting their secretion of pro-inflammatory, angiogenic and immunoregulatory factors, therefore, contributing to tumor progression and metastasis (109–112). Overall, the increased upregulation of CXCL9 and CXCL10, accompanied with the downregulation in CXCL2, may underlie the observed alterations in the cellular components at the level of systemic organs and within the tumor microenvironment following combination treatment. In the current study, gene expression analysis was carried out on whole tumor tissue, which limits the interpretation of our data. Given that the tumor microenvironment is very heterogenous, in addition to the fact that cytokines and chemokines are differentially secreted and have distinct functions, it would be advantageous if gene expression analysis was performed on purified subsets of intratumoral immune cells. This would lead to a more accurate understanding of the interplay between immune cells and the tumor tissue.

**Figure 12:**
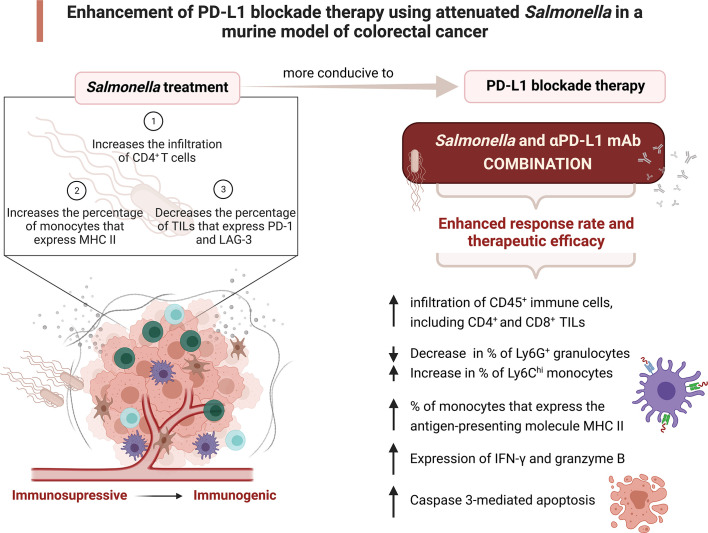
Attenuated *Salmonella* enhances the therapeutic efficacy of PD-L1 blockade in a murine colon adenocarcinoma model. A low dose of attenuated *Salmonella* transforms the tumor microenvironment from being immunosuppressive to become immunogenic. *Salmonella*-induced alterations in the intratumoral immune system components enhances the response rate and therapeutic outcome of PD-L1 blockade. The potential underlying mechanisms of the improved effect of combination treatment are illustrated. Created with BioRender.com.

In addition to the immunomodulatory activity, our investigation provides a novel insight into another mechanism by which the combination treatment operates. Caspase-3 plays an essential role in inducing apoptosis through both the intrinsic and extrinsic pathways and the presence of cleaved caspase 3 in cells is a marker for apoptosis. The level of cleaved caspase 3-positive cells was higher in tumors from mice treated with a low dose of attenuated *Salmonella* compared to untreated control mice. The induction of apoptosis through caspase 3 activation post *Salmonella* treatment was previously demonstrated by other groups (113, 114). The present study revealed that the combined treatment enhanced caspase 3-mediated apoptosis to a greater extent compared to monotherapy, and this highlights a potential mechanism by which tumor growth is controlled. The increase in caspase-3 mediated apoptosis could be related to the upregulated expression of granzyme B post treatment with *Salmonella* and αPD-L1 combined (115). **Figure 12** summarizes the major findings of the current study.

Taken altogether, this study has elucidated new insights into modulating the microenvironment of colorectal cancer using attenuated *Salmonella*. The findings highlight the possibility of *Salmonella* treatment to alter the phenotypic and functional characteristics of intratumoral immune cells in order to enhance the efficacy and response rate to PD-L1 blockade. This forms a rational basis for further exploration of using *Salmonella* plus αPD-L1 combination to enhance the therapeutic outcome of ICIs in colorectal cancer. Recognizing that the findings of the current study are limited to the murine MC38 colon adenocarcinoma model, additional work is needed to confirm the improved effect of the proposed immune-based combination treatment against a broad-spectrum of cancer types that differ in their immunogenicity, tumor microenvironment complexity and mechanisms of resistance to immunotherapy.

## Data availability statement

The original contributions presented in the study are included in the article/**Supplementary Material**. Further inquiries can be directed to the corresponding author.

## Ethics statement

The animal study was reviewed and approved by The animal research ethics committee of the College of Medicine and Health Sciences, UAE University (#ERA_2018_5743).

## Author contributions

BA-R conceived the study and designed the experiments. BA-S, AA-S, GB, YM and RM conducted experiments. BA-S acquired data. BA-S, MF-C and BA-R analyzed experimental data. BA-S and BA-R wrote the manuscript. MF-C provided scientific insights and edited the manuscript. BA-R supervised the project. All authors contributed to the article and approved the submitted version.
