# Supplementary material for: Attenuated Salmonella potentiate PD-L1 blockade immunotherapy in a preclinical model of colorectal cancer
Source: Front Immunol. 2022 Dec 20;13:1017780. doi: 10.3389/fimmu.2022.1017780 (PMC9807881; doi:10.3389/fimmu.2022.1017780)
Supplement: Supplementary file 1 [file DataSheet_1.pdf]

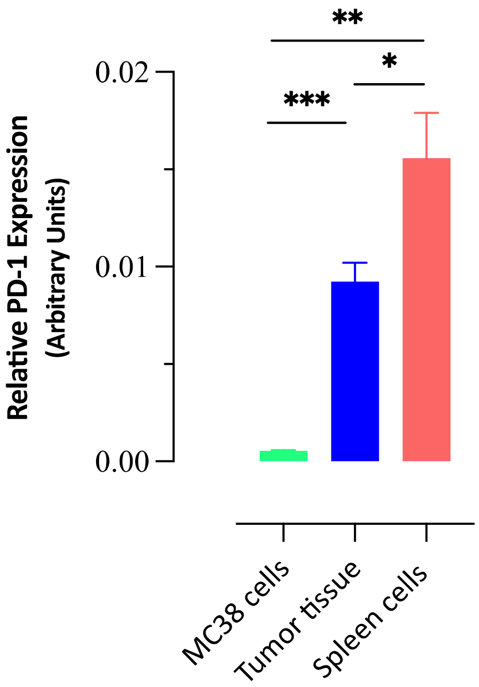

Supplementary Figure 1: Relative expression levels of PD-1 mRNA isolated from in vitro grown MC38 cells (n=3), whole MC38 tumor tissue (n=8), or normal spleen cells (n=6). Asterisks denote statistically significant differences between the indicated groups (***p < 0.001; **p < 0.01; *p < 0.05). The results are pooled from three independent experiments.

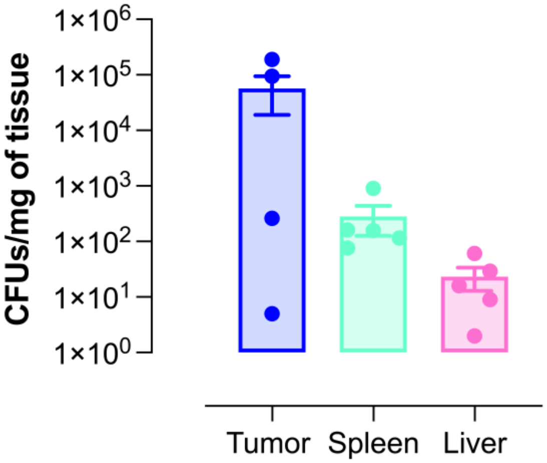

Supplementary Figure 2: Bacterial colonization in different organs in MC38 tumor-bearing mice. MC38 tumor cells (2×10^5) were subcutaneously implanted into the right flank of C57BL/6 mice. 14 days post implantation, mice were injected intraperitoneally with BRD509E (~5×10^3 CFUs). Tumor-bearing mice were sacrificed on day 19 following treatment, organs were resected and bacterial loads (as described in the materials and methods) were determined in terms of CFUs/mg of tissue. The data shown is from one independent experiment.

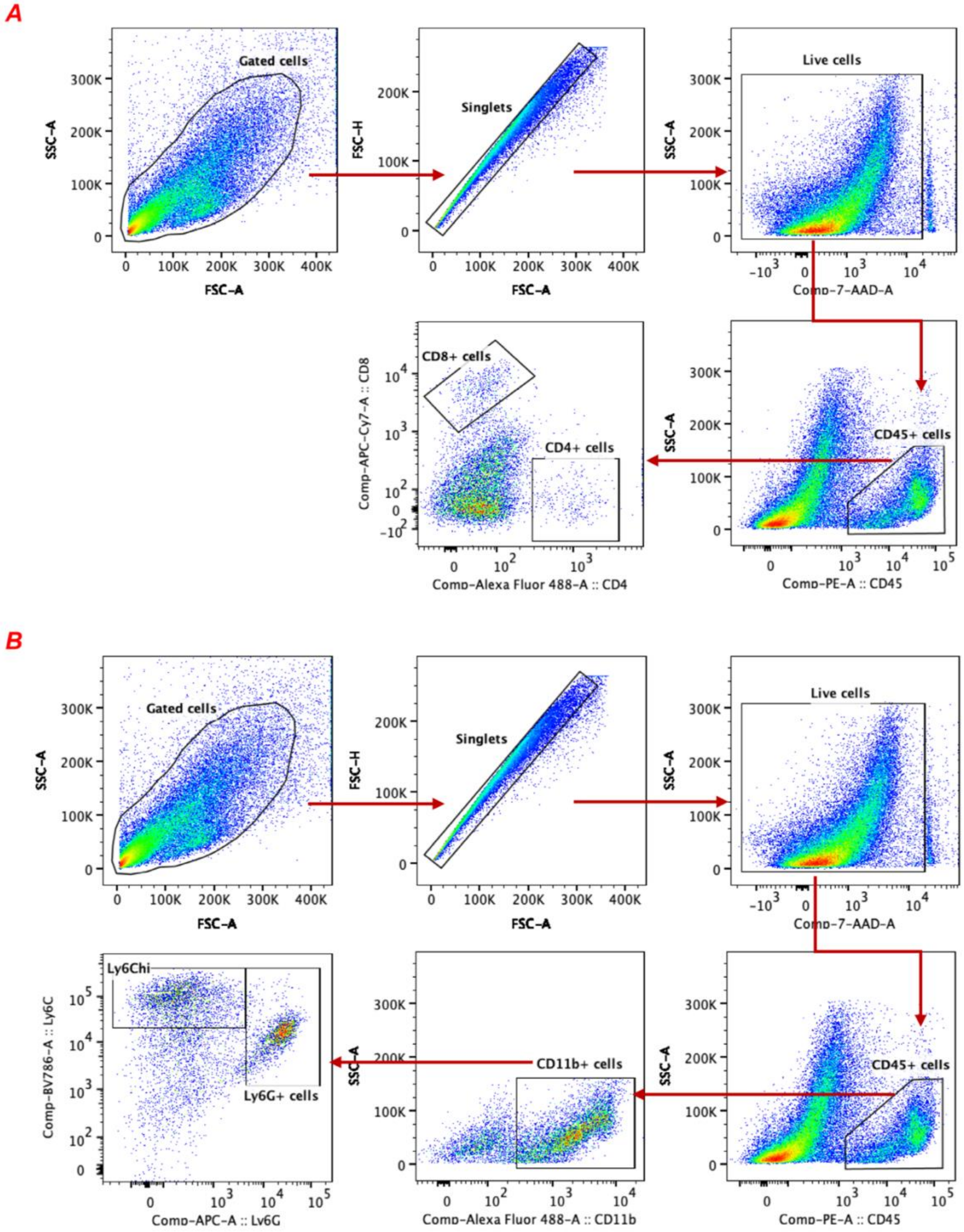

Supplementary Figure 3: Gating strategies used to identify intratumoral lymphoid and myeloid subsets. ***(A)*** Lymphoid subpopulations were identified using an antibody staining panel that include: CD45-PE, CD4-Alexa Flour 488, CD8-APC-Cy7 and 7AAD viability dye. ***(B)*** Myeloid subpopulations were identified using another antibody panel that include: CD45-PE, CD11b-Alexa Fluor 488, Ly6G-APC, Ly6C-BV785 and 7AAD viability dye.

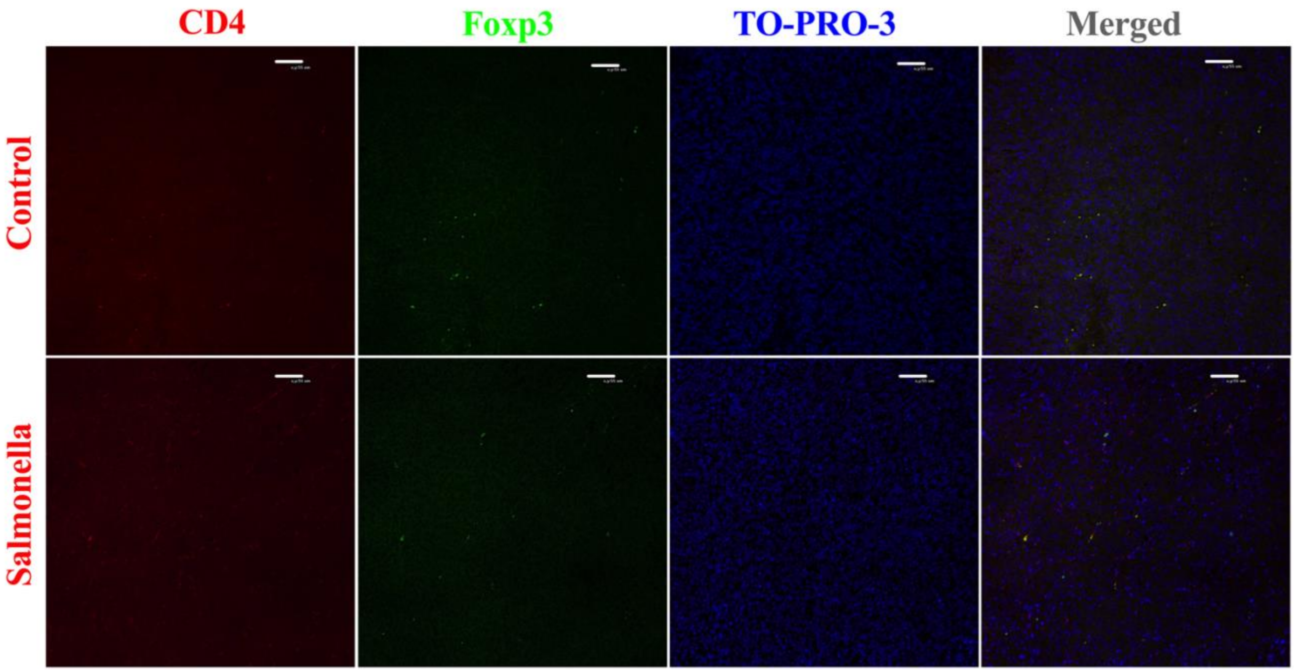

Supplementary Figure 4: Treatment with a low dose of *Salmonella* decreased Tregs/CD4$^+$ cells ratio in MC38 tumor tissues. Tumor sections were stained with anti CD4 and anti Foxp3 antibodies as described in the material and methods section. Representative immunofluorescent images- at lower magnification- of CD4, Foxp3, TO-PRO-3 nuclear staining and the merge picture from the control and *Salmonella*-treated groups are presented. Scale bar 55 µm.

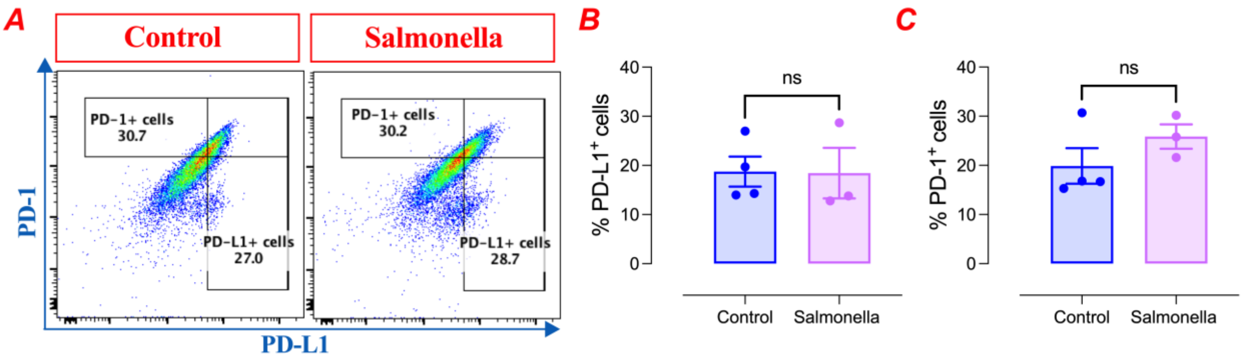

Supplementary Figure 5: *Salmonella* treatment did not alter the expression of PD-L1 or PD-1 on CD45$^-$ cells in MC38 tumor tissues. MC38 tumors were harvested from tumor-bearing mice on day 14 post bacterial treatment and CD45$^-$ cells were analyzed for their expression of PD-L1 or PD-1. Representative dot plots ***(A)*** and combined results analysis of the percentage of CD45$^-$ cells that express PD-L1 ***(B)*** or PD-1 ***(C)*** are illustrated for the control and *Salmonella*-treated groups. The data is presented using mean ± SEM of 7-8 mice per group pooled from two independent experiments.

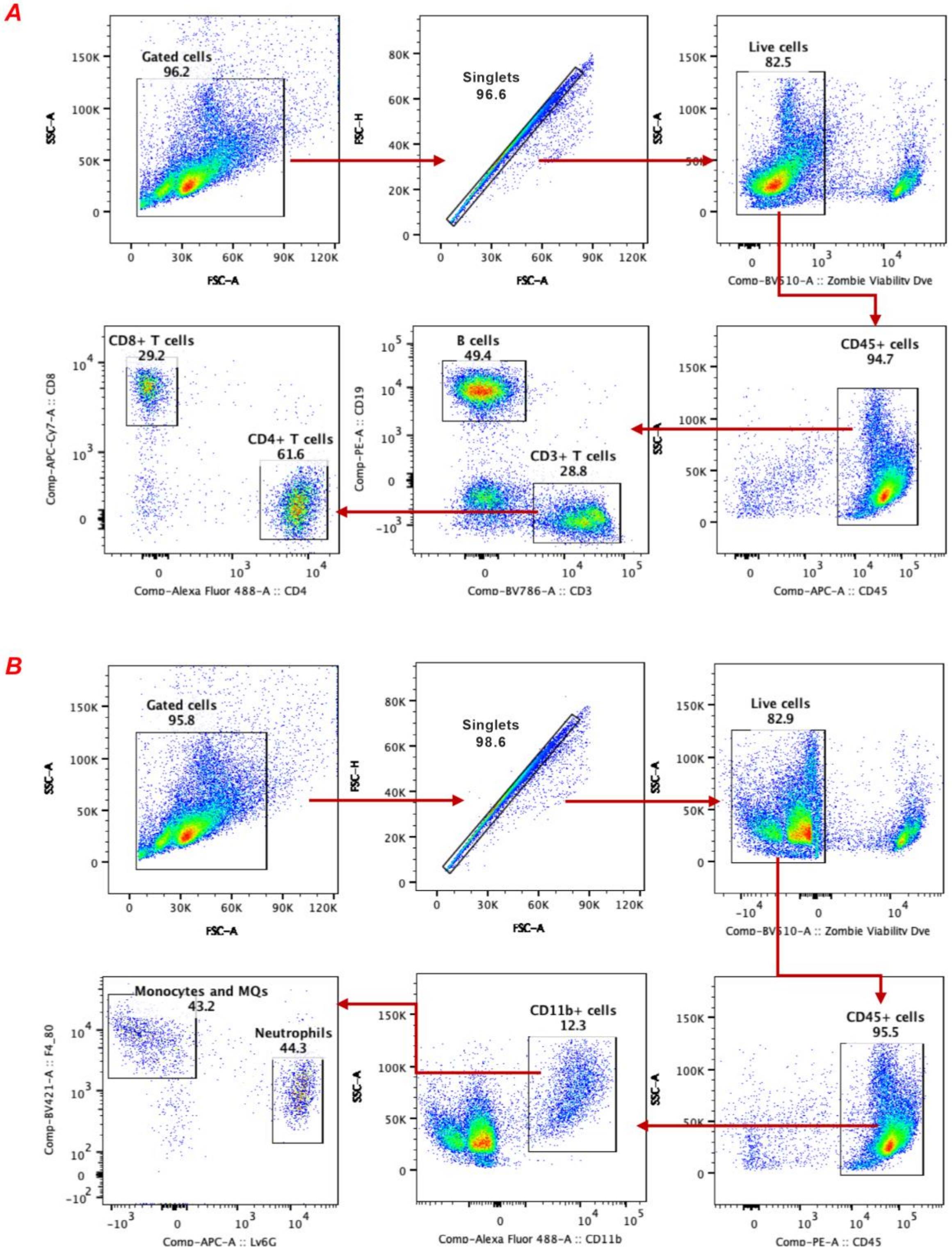

Supplementary Figure 6: Gating strategies used to identify the splenic lymphoid and myeloid subsets. ***(A)*** Lymphoid subpopulations were identified using an antibody staining panel that include: CD45-APC, CD3-BV 785, CD19-PE, CD4-FITC, CD8-APC-Cy7, and Zombie Aqua viability dye. ***(B)*** Myeloid subpopulations were identified using another antibody panel that include: CD45-PE, CD11b-Alexa Fluor 488, Ly6G-APC, F4/80-BV 421 and Zombie Aqua viability dye.

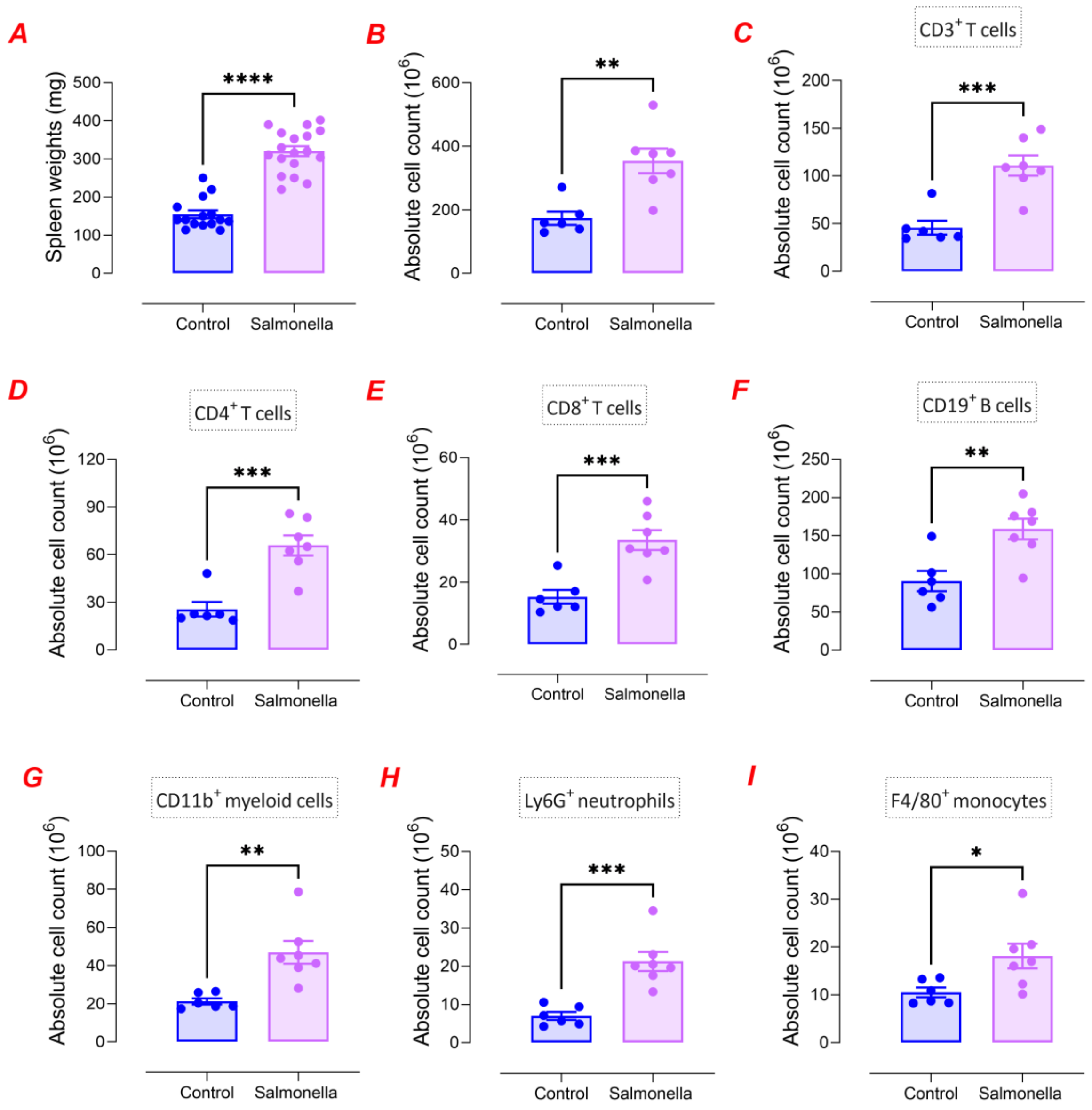

Supplementary Figure 7: *Salmonella* increases the number of lymphoid and myeloid immune cells in the spleens of MC38 tumor-bearing mice. The effect of *Salmonella* treatment on spleen weights is illustrated **(A)**. The absolute cell counts of total spleen ***(B)***, T cells ***(C)***, CD4$^+$ T cells ***(D)***, CD8$^+$ T cells ***(E)***, B cells ***(F)***, CD11b$^+$ myeloid cells ***(G)***, neutrophils (Ly6G$^+$) ***(H)*** and monocytes/macrophages (Ly6G$^-$ F4/80$^+$) ***(I)*** were determined and presented. Each data point represents a single mouse pooled from two independent experiment. Asterisks denote statistically significant differences from control group, * ($P < 0.05$), ** ($P < 0.01$), *** ($P < 0.001$) and ns (no statistical significance, > 0.05).

Table I

Experimental Group	Rate Constant (k) (95% CI)	Doubling Time (days) (95% CI)
Control (Saline)	0.1835 (0.1279 to 0.2621)	3.777 (2.645 to 5.418)
Control (Isotype Ab)	0.1823 (0.1393 to 0.2378)	3.803 (2.915 to 4.975)
Salmonella Only	0.1679 (0.1257 to 0.2226)	4.129 (3.113 to 5.515)
Salmonella + IgG	0.1634 (0.0556 to 0.4063)	4.242 (1.706 to 12.46)
Anti-PD-L1 Ab Only	0.1752 (0.0931 to 0.3194)	3.957 (2.170 to 7.444)
Salmonella+anti-PD-L1 Ab	0.1091 (0.0766 to 0.1475)	6.356 (4.699 to 0.045)
